# Pleistocene dynamics of the Eurasian steppe as a driving force of evolution: Phylogenetic history of the genus *Capsella* (Brassicaceae)

**DOI:** 10.1002/ece3.8015

**Published:** 2021-08-18

**Authors:** Anže Žerdoner Čalasan, Herbert Hurka, Dmitry A. German, Simon Pfanzelt, Frank R. Blattner, Anna Seidl, Barbara Neuffer

**Affiliations:** ^1^ Department 5: Biology/Chemistry, Botany University of Osnabrück Osnabrück Germany; ^2^ South‐Siberian Botanical Garden Altai State University Barnaul Russia; ^3^ Experimental Taxonomy Leibniz Institute of Plant Genetics and Crop Plant Research (IPK) Seeland‐Gatersleben Germany; ^4^ Munich Botanical Garden München Germany; ^5^ Institute of Botany Department of Integrative Biology and Biodiversity Research University of Natural Resources and Life Sciences Vienna (BOKU) Austria

**Keywords:** evolution, hybridization, introgression, refugium, shepherd's purse

## Abstract

*Capsella* is a model plant genus of the Brassicaceae closely related to *Arabidopsis*. To disentangle its biogeographical history and intrageneric phylogenetic relationships, 282 individuals of all five currently recognized *Capsella* species were genotyped using a restriction digest‐based next‐generation sequencing method. Our analysis retrieved two main lineages within *Capsella* that split *c*. one million years ago, with western *C. grandiflora* and *C. rubella* forming a sister lineage to the eastern lineage consisting of *C. orientalis*. The split was attributed to continuous latitudinal displacements of the Eurasian steppe belt to the south during Early Pleistocene glacial cycles. During the interglacial cycles of the Late Pleistocene, hybridization of the two lineages took place in the southwestern East European Plain, leading to the allotetraploid *C. bursa‐pastoris*. Extant genetic variation within *C. orientalis* postdated any extensive glacial events. Ecological niche modeling showed that suitable habitat for *C. orientalis* existed during the Last Glacial Maximum around the north coast of the Black Sea and in southern Kazakhstan. Such a scenario is also supported by population genomic data that uncovered the highest genetic diversity in the south Kazakhstan cluster, suggesting that *C. orientalis* originated in continental Asia and migrated north‐ and possibly eastwards after the last ice age. Post‐glacial hybridization events between *C. bursa‐pastoris* and *C. grandiflora/rubella* in the southwestern East European Plain and the Mediterranean gave rise to *C. thracica*. Introgression of *C. grandiflora*/*rubella* into *C. bursa‐pastoris* resulted in a new Mediterranean cluster within the already existing Eurasian *C. bursa‐pastoris* cluster. This study shows that the continuous displacement and disruption of the Eurasian steppe belt during the Pleistocene was the driving force in the evolution of *Capsella*.

## Graphical abstract

*Capsella* is a model plant genus of the Brassicaceae, closely related to *Arabidopsis*. We present here the evolutionary history of the genus *Capsella* placed into space and time.

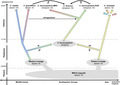

## 1 INTRODUCTION

*Capsella* Medik. is a small genus within the mustard family (Brassicaceae). Of all wild relatives of the model system *Arabidopsis* (DC.) Heynh., *Capsella* is the most closely related and intensively investigated genus. Both genera belong to the tribe Camelineae, which also includes *Camelina* Crantz and four more genera (https://brassibase.cos.uni‐heidelberg.de). The three named genera have become model systems for plant molecular research, as well as the study of genome evolution, and the evolution and development of genotypic and phenotypic traits.

Species delimitation within *Capsella* is difficult because of the enormous morphological variation especially in leaf and fruit characters, and in hybridization potential studied intensively as early as in the 1900s (Almquist, 1907; Shull, 1909). In most cases, five species are accepted (Chater in Flora Europaea 1964; Koch et al., 2018): *Capsella grandiflora* (Fauché & Chaub.) Boiss., *C. rubella* Reut., *C. orientalis* Klokov, *C. bursa‐pastoris* (L.) Medik., and *C. thracica* Velen. Less often, some of the species are treated at infraspecific rank, for example, *C. thracica* (Chater, 1993; Stojanov & Stefanov, 1948) or *C. orientalis* (Tzvelev, 2000a) as a subspecies of *C. bursa‐pastoris*. An ultimately lumping approach recognizes the genus as monotypic, comprising the five abovementioned species (Appel & Al‐Shehbaz, 2003; Svensson, 1984). The wealth of available morphological and molecular evidences supports acceptance of the above five entities as distinct species. The first three species are diploid (2n = 2x = 16), and the latter two are tetraploid (2n = 4x = 32). *Capsella grandiflora* is an obligate outcrossing species with a sporophytic self‐incompatibility system (Paetsch et al., 2006; Riley, 1936), whereas all other species are predominantly selfing.

The evolutionary history of the genus *Capsella* has been controversial for a long time. However, by including the commonly neglected species *C. orientalis* and *C. thracica* in molecular phylogenetic analyses, Hurka et al. (2012) discovered the “missing link” in the understanding of the origin of tetraploid *C. bursa‐pastoris* and provided evidence that in *Capsella* the transition from outbreeding to selfing at the diploid level occurred twice independently. This was later confirmed by several other studies, for example, Douglas et al. (2015), Bachmann et al. (2019), and Žerdoner Čalasan et al. (2019). The most recent common ancestor (MRCA) of *Capsella* is presumably of Late Pliocene/Early Pleistocene age and was probably distributed along the Eurasian steppe belt from eastern Europe to western or even Central Asia (Hurka et al., 2012). During the Pleistocene, *Capsella* split into two lineages, the eastern and the western lineage. The eastern lineage comprises the extant species *Capsella orientalis* and the western lineage the extant species *Capsella grandiflora* and *C*. *rubella*. *Capsella bursa‐pastoris* and *C. thracica* emerged from hybridization between the western and eastern lineages. Meanwhile, *Capsella* has become a model system for genomic studies of plant mating systems (e.g., Bachmann et al., 2019, 2021; Brandvain et al., 2013; Mattila et al., 2020; Woźniak et al., 2020) and polyploidization (Douglas et al., 2015; Han et al., 2015; Kryvokhyzha, Salcedo et al., 2019; Omelchenko et al., 2020). In contrast to the increasing knowledge about *Capsella* at the genomic level, a detailed phylogeographical aspect of its evolution, especially of the eastern lineage consisting of *C. orientalis*, is currently missing. *Capsella orientalis* was first described by Klokov in 1926 and for decades eked out a niche existence in botanical research. While the first indications of its diploid nature are dated to the early 2000s (Dorofeyev, 2002; Tzvelev, 2000b), it was only in 2012 when this species was unambiguously placed into the wider evolutionary time frame of *Capsella* and its distribution range properly delimitated (Hurka et al., 2012).

*Capsella orientalis* ranges from southeastern Europe, the South Urals, northern and eastern Kazakhstan, and southwestern Siberia to the northern part of the Chinese Xinjiang province and Mongolia. Its distribution thus coincides with the Eurasian steppe belt, namely its Euro‐Siberian part (Hurka et al., 2012). Prototypes of the Eurasian steppe appeared during the Lower Miocene (23–16 Ma) in Central Asia. Continuous global cooling through the Middle and Upper Miocene accompanied by the uplift of surrounding mountain ranges and the retreat of water bodies have initiated and facilitated the aridification of Central Asia (Hurka et al., 2019 and references therein; Barbolini et al., 2020). This in turn promoted the expansion of drought‐adapted steppe forbs and grasses. Spreading toward the west, the Eurasian steppe belt reached the East European Plain in Late Miocene (Akhmetyev et al., 1999; Velichko, 1999; Velichko et al., 2005; 2011b) and expanded in Eastern Europe during the Pliocene (Lang, 1994; Mai, 1995). The Eurasian steppe belt was under strong influence of the Pleistocene glaciations that caused extensive expansions and contractions as well as latitudinal shifts and longitudinal splits of the ranges of steppe plant species during the past 3 million years (Hurka et al., 2019 and references therein). It has been shown that such a biome dynamic is mirrored by molecular signals of typical Eurasian steppe plants (e.g., *Capsella orientalis*) and thus reflects their biogeographical history (Buono et al., 2021; Franzke et al., 2004; Friesen et al., 2016, 2020; Hantemirova et al., 2020; Hurka et al., 2012; Seidl et al., 2019, 2021; Seregin et al., 2015; Volkova et al., 2017).

Using a restriction digest‐based NGS method of genotyping‐by‐sequencing (GBS) on a representative taxon sample of 282 *Capsella* individuals, we investigated whether and how the Pleistocene glaciations influenced the short but turbulent history of the genus *Capsella*. Furthermore, in accord with Fernández‐Mazuecos et al. (2018) we tested to what extent filtering and SNP calling may influence the retrieved topologies and admixture as well as genetic clustering of tested populations. To investigate whether Pleistocene glaciations influenced the genetic structure of the steppe element *C. orientalis*, we calculated *F*‐statistics and molecular diversity indices, and conducted an analysis of molecular variance (AMOVA). While Altai has been commonly recognized as a refugial area for steppe flora and fauna, we wanted to assess via ecological niche modeling whether this mountain range had the same refugial role in case of younger taxa, such as *C. orientalis*.

## 2 MATERIALS AND METHODS

**FIGURE 1:**
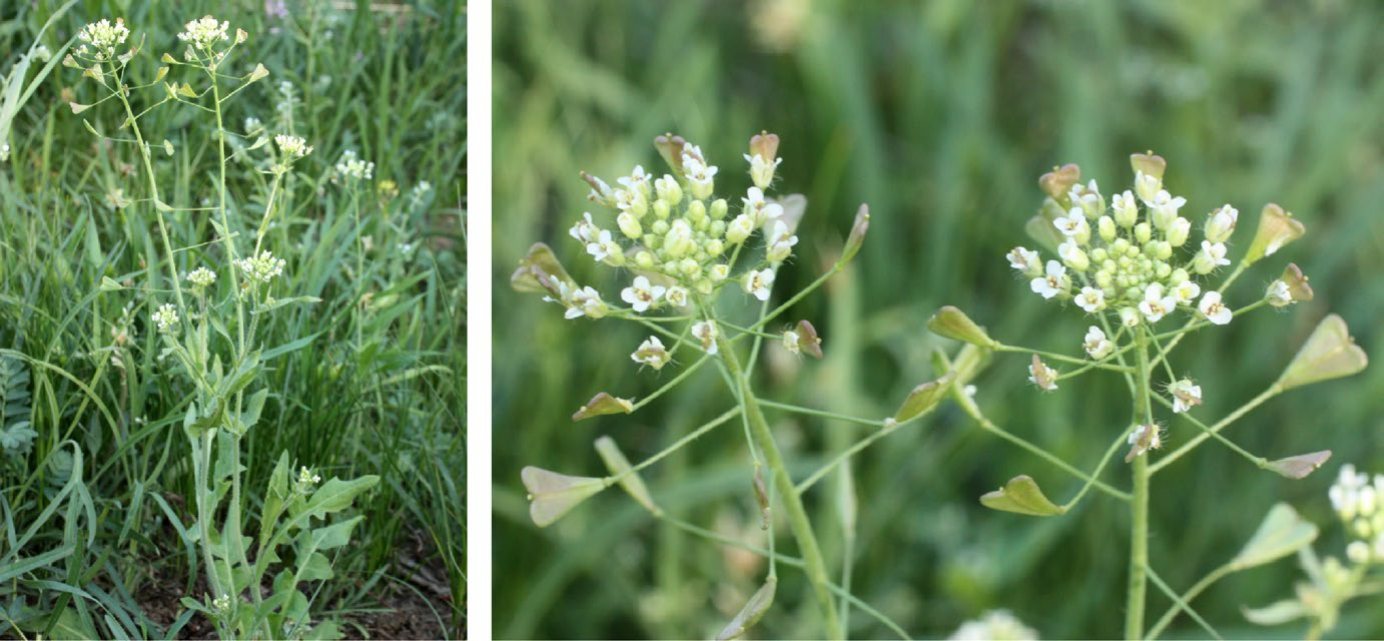
Habitus of *Capsella orientalis* Klokov. Whole plant (left) and inflorescence (right) of *Capsella orientalis* (Astrakhan region, Bogdinsko‐Baskunchaksky nature reserve, Bolshoy Bogdo, cereal‐forb steppe at the southern foot of the mountain; 4 May 2011; by M. S. Knyazev)

Seeds of 5–10 randomly selected plants per population collected in the wild (Figure S1) were cultivated in the greenhouse of the University of Osnabrück, Germany (Figure 1). We investigated 18 populations of *Capsella bursa‐pastoris*, seven populations of *C. grandiflora*, 57 populations of *C. orientalis*, five populations of *C. rubella*, and four populations of *C. thracica*.

Plants were grown to the flowering stage and ploidy levels were estimated through flow cytometry to minimize species determination errors. Genome size estimations followed the protocols described in Doležel et al. (2007) with some modifications: 1 cm^2^ of young fresh leaf tissue was chopped together with 1 cm^2^ of tissue from the internal standard and 0.4 ml of cold CyStain® Nuclei Extraction Buffer in a glass petri dish with a razor blade. The cell suspension was vortexed briefly and then stained with 1.6 ml CyStain® Staining Buffer containing 4’,6‐diamidino‐2‐phenylindole (DAPI). The samples were incubated for 30 s, mixed gently, and filtered through a 50 μm CellTrics® filter. Flow cytometry was performed with the CyFlow® Ploidy Analyser Typ 24, 2010 (Sysmex Partec GmbH, Germany) with the following settings: gain: 540 V, velocity: 0.4 μl/s, 365 nm UV‐LED, 532 nm excitation, and 532 nm emission. Samples were run until 5,000 cells per peak were measured with each measurement repeated thrice (CV < 5%). *Petroselinum crispum* (Mill.) Fuss served as an internal standard (4.46 pg/2C, *SD* ± 0.08 pg/2C: Yokoya et al., 2000) for diploid as well as for polyploid species. Up to 1 g of fresh leaves were silica‐dried and stored at room temperature. Subsequent DNA isolation was carried out using DNeasy Plant Mini Kit (Qiagen), following the manufacturer's protocol.

Genotype‐by‐sequencing library preparation followed protocols from Poland et al. (2012), and Meyer et al. (2008). We performed single‐end sequencing of 100 bp fragments of 282 samples (57 populations) in two runs on a HiSeq 2500 (Illumina, CA, USA) at the Leibniz Institute of Plant Genetics and Crop Plant Research (Gatersleben, Germany). To allow for representative coverage, the sequenced DNA amount of tetraploid *C. bursa‐pastoris* and *C. thracica* accessions was doubled. To assess the reproducibility of the sequencing method internally, three samples were loaded twice on the flow cell (depicted with * in Figure S1).

### 2.1 SNP filtering/calling

Already demultiplexed raw Illumina DNA sequence data files were analyzed using *ipyrad* v0.9.52 (Eaton & Overcast, 2020). Two separate sub‐datasets were analyzed, one containing all *Capsella* species (282 accessions; caps dataset) and one encompassing only *C. orientalis* accessions (235 accessions; ori dataset). Although there are already published reference genomes of *C. rubella* and *C. grandiflora* (Slotte et al., 2013) available, we assembled both of the sub‐datasets de novo to prevent SNP (single nucleotide polymorphism) recovery bias. Several analyses were run to test the influence of different parameter settings on the final SNP data matrix. The clust_threshold option was set in parallel to 0.85, 0.90, and 0.95, recognizing reads as homologous if the similarity exceeded 85%, 90% and 95%, respectively. The min_sample_locus was set in a way that only sites with at least 50%, 25%, or 12.5% of data were retained in the final alignment, respectively. The *Capsella* dataset consisted of two tetraploid species; thus, the max_alleles_consens option was set to 4, while this parameter was left at the default value of 2 for the analysis of the *Capsella orientalis* dataset consisting of purely diploid accessions. After initial testing, we set the max_SNPs_locus parameter to 0.1 and 0.05, respectively, in the most stringently filtered datasets (i.e., 0.95 with at least 50% of SNP data per position). The default value of 0.2, indicating that a locus might consist of up to 20% of SNPs, might be set inappropriately high for such a young plant taxon. In total, we analyzed 22 different datasets. All subsequent analyses were carried out using the most stringently filtered datasets with the clustering threshold set to 95%, including SNP positions with at least 50% of available information and allowing for a SNP maximum of 5% per locus.

### 2.2 Phylogenetic inference

We performed a SVDQuartets analysis (Chifman & Kubatko, 2014) in PAUP* v4.0a (Swofford, 2002) first using all 282 *Capsella* accessions (unreduced SVDQ dataset). However, due to extremely high admixture recovered from the population genetic structure analyses, another analysis was carried out including only accessions showing more than 95% association to a specific cluster (i.e., exhibiting <5% admixture), reducing the dataset from 282 accessions to 57 (reduced SVDQ dataset). To verify the monophyly of species, no accession‐to‐species attribution was carried out prior to the analyses. We evaluated all possible quartets in the reduced dataset and 10,000 quartets in the unreduced one. The handling of the ambiguities was set to “distribute.” QFM was specified as the quartet assembly algorithm. Bootstrapping was performed with the number of replicates set to 1,000. Final trees were exported with saved internal nodes and visualized with FigTree v1.4.3 (http://tree.bio.ed.ac.uk/software/figtree/).

### 2.3 Time divergence estimation

Time divergence analysis was carried out in SNAPP (Bryant et al., 2012) coupled with a molecular clock model (Stange et al., 2018), which is implemented as an add‐on package in BEAST2 (Bouckaert et al., 2019). The SNAPP input file was generated using a ruby script developed by M. Matschiner (available at: https://raw.githubusercontent.com/mmatschiner/snapp_prep/master/snapp_prep.rb). An unlinked *Capsella* SNP dataset generated from ipyrad was used as an input file; however, due to the high computational intensity, further reduced to 25 accessions, including all major taxon groups as retrieved in the SVDQ analysis. The 25 accessions were selected so that the genetically most divergent accessions within selected species and/or genotypes were represented, covering the whole currently known distribution area. We provided a newly reconstructed starting tree from an SVDQ analysis but without a specified out‐group to avoid artificial topologies. We made use of four different secondary calibration points that have been independently inferred from the following unrelated studies using different calibration methods, different taxon samples, and different loci: Douglas et al., 2015, Han et al., 2015, Hurka et al., 2012 and Žerdoner Čalasan et al., 2019. The calibration node ages were specified as log‐normally distributed in real space. We performed 20,000,000 Markov‐chain Monte Carlo (MCMC) iterations per SNAPP analysis, and the stationarity of MCMC chains was assessed using Tracer v1.7.0 (Rambaut et al., 2018). Trees generated in three independent analyses were combined in LogCombiner v.2.5.2 with an additional burn‐in of 10%, and the final tree was generated using TreeAnnotator v2.5.2 using the “maximum clade credibility tree with median heights” option. All phylogenetic analyses were carried out at the CIPRES Science Gateway computing facility (Miller et al., 2010).

### 2.4 Inference of genetic structure

Population genetic structure was assessed using the clustering algorithm implemented in STRUCTURE v.2.3.4 (Pritchard et al., 2000) by carrying out a run with length of 10,000 MCMC iterations with a burning period of 2,000. For the *Capsella* dataset, we tested a range of *K*‐values from *K* = 2 to *K* = 10 and repeated the analysis 20 times. For the *Capsella orientalis* dataset, we tested a range of *K*‐values from *K* = 2 to *K* = 5 with the same number of iterations as specified above. All settings were left at default with the exception of the correlated allele frequencies and admixture options. Due to previous experience, we expected notable introgression. Consistency across replicate cluster analyses was assessed with CLUMPP v.1.1.2. (Jakobsson & Rosenberg, 2007) by using Greedy algorithm and 1,000 random input orders. *K* probability was assessed using the Evanno method (Evanno et al., 2005) as implemented in Structure Harvester (Earl & von Holdt, 2012). Population specific ancestry coefficients were calculated as mean values of the individuals and shown as pie charts plotted on the map. In addition, population genetic structure was also inferred using a non‐Bayesian clustering algorithm, namely sparse non‐negative matrix factorization as implemented in the R package LEA (an R package for Landscape and Ecological Association Studies: Frichot & François, 2015; R Core Team, 2013). Twenty hypothetical ancestral populations were tested with 50 repetitions each for both datasets, and the *K* probability was assessed using the cross‐entropy approach incorporated in the LEA package. Population specific ancestry coefficients were calculated and depicted as specified above. To confirm the result, additional DAPC analyses for the *Capsella* as well as *C. orientalis* datasets were carried out under default parameters using the adegenet R package (Jombart, 2008).

### 2.5 Ecological niche modeling and assessment of the geographical range

**FIGURE 2:**
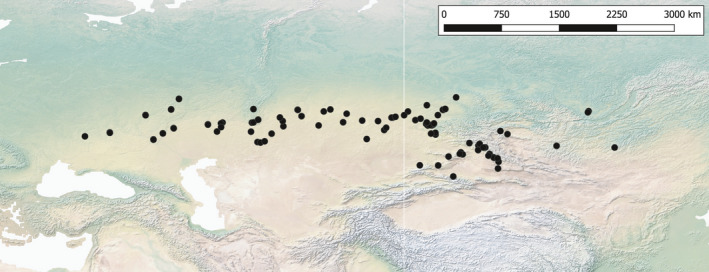
Distribution map of *Capsella orientalis* based on geographical range information from the literature (Ebel, 2002; German & Ebel, 2009; German et al., 2012; German, 2015; Han et al., 2015; Hurka et al., 2012; Iljinska et al., 2007; Jalas & Suominen, 1994; Kryvokhyzha, Salcedo et al., 2019; Kulikov, 2005; Sheremetova et al., 2011; Verkhozina et al., 2020), our own field data, and a priori critically assessed GBIF collection points

To infer the climatic suitability of habitats of *Capsella orientalis* through space and time, ecological niche modeling was employed and implemented in MaxEnt v3.3.4 (Phillips et al., 2006). A set of 19 bioclimatic variables were retrieved from the WorldClim database (http://worldclim.com/paleo‐climate1: Hijmans et al., 2005) at a spatial resolution of 2.5 arc minutes (approximately 4.5 km^2^ at the equator) and for three different time slices: present, Mid Holocene (8.33–4.2 ka), and Last Glacial Maximum (ca. 21 ka). The geographical distribution of *Capsella orientalis* was assessed based on geographical range information from the literature (Ebel, 2002; German, 2015; German et al., 2012; German & Ebel, 2009; Iljinska et al., 2007; Jalas & Suominen, 1994; Kulikov, 2005; Sheremetova et al., 2011; Verkhozina et al., 2020), Global Biodiversity Information Facility (GBIF: https://www.gbif.org), and herbarium and own field data. The final dataset consisted of 121 entries (Figure 2 and Figure S2). Variable exclusion via cross‐correlation was carried out to assure that the used bioclimatic variables were uncorrelated (Pearson's correlation coefficient <0.5). Selected variables were bio4 (temperature seasonality), bio5 (maximum temperature of the warmest month), and bio12 (annual precipitation). To maximize predictive ability and avoid model overfitting, the number of climate variables was kept to a minimum. Models were constructed using Maxent v3.4.1 (Phillips et al., 2006) and evaluated with the R package ENMeval (Muscarella et al., 2014).

### 2.6 Population genetics analysis

Arlequin v3.5 (Excoffier & Lischer, 2010) was used to determine the genetic diversity of *Capsella orientalis* populations, calculate *F*‐statistics and distribution of private alleles, and assess the genetic differentiation on the cluster‐ as well as population‐level via AMOVA (Analysis of molecular variance). Molecular diversity indices and private allele sharing were assessed with the complete ori SNP dataset retrieved directly from the ipyrad analysis, while F‐statistics and AMOVA were carried out on a *.loci‐dataset also retrieved directly from the ipyrad workflow. The significance of the test results was based on 1,023 permutations. We categorized the populations into five genetic clusters corresponding to the ancestral populations inferred from the LEA analysis. For AMOVA, we used only five populations per genetic cluster/geography, as this was the lowest possible number of investigated populations per genetic cluster/geographical region (only five populations belonged to the Central Asian genetic cluster/region). Five geographically most distant populations were selected for genetic clusters that consisted of more than five populations.

## 3 RESULTS

### 3.1 SNP filtering/calling

The final analyses were carried out using the *Capsella* (caps) dataset comprising 282 accessions representing different *Capsella* species and *Capsella orientalis* (ori) dataset comprising 235 accessions of *C. orientalis*. Furthermore, the reads had to be at least 95% similar to be recognized as homologous and only positions where at least 50% of samples had a recorded base were retained in the final alignment. Maximum percentage of allowed SNPs per locus retained in the final alignment was set to 5%. Combination of these parameters consistently outperformed other parameter assemblages (Figure S3), in which no population structure could have been inferred throughout different *K*‐values (data not shown). The final *Capsella* dataset consisted of 12,635 SNPs and 13.17% missing sites, while the final *Capsella orientalis* dataset included 4,644 SNPs with 24.23% of missing data. The alignment used for time divergence estimation consisted of 25 entries, 16,398 SNPs (out of which 6,320 were retrieved in the unlinked SNP dataset) and 20.07% missing sites. Further statistics can be inferred from the File S2.

### 3.2 Phylogenetic inference

**FIGURE 3:**
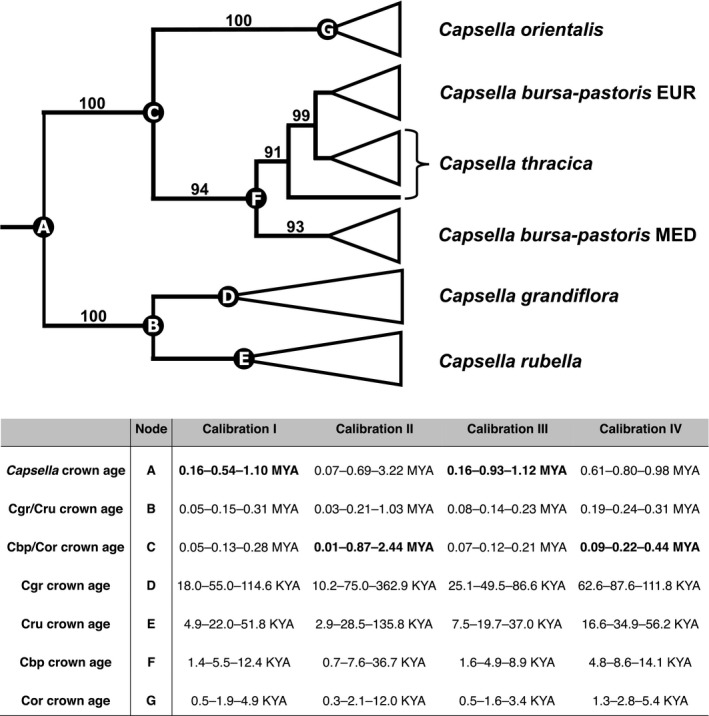
Above: Schematic representation of phylogenetic relationships in the genus *Capsella*. Phylogenetic reconstruction is based on a coalescent SVDQ algorithm analysis of all 282 accessions (shown in full in File S3). The values above branches represent bootstrap support values (values below 90 not shown). The node letters correspond to the letters in the table below. Below: Time spans retrieved from SNAPP analyses based on different calibration points. Time spans are represented as median node ages with minimum and maximum age span corresponding to the 95% HPD. The age spans in bold represent the individual calibration points for the time divergence analyses and are retrieved from the following studies: calibration I (Žerdoner Čalasan et al., 2019), calibration II (Hurka et al., 2012), calibration III (Douglas et al., 2015), and calibration IV (Han et al., 2015). Abbreviations: Cgr *Capsella grandiflora*; Cru *Capsella rubella*; Cbp *Capsella‐bursa‐pastoris*; Cor *Capsella orientalis*

The SVDQ analysis of the nonreduced dataset (Figure S4) retrieved only poorly supported species‐specific clades of *Capsella rubella* (57 BS) and *C. grandiflora* (37 BS) but well‐supported species‐specific clades of *C. orientalis* (100 BS) and *C. bursa‐pastoris* (94 BS). Within the latter, the Mediterranean *C. bursa‐pastoris* was monophyletic (93 BS), while the Eurasian *C. bursa‐pastoris* and *C. thracica* were retrieved as poorly supported clade and grade, respectively (Figure S4). *C. grandiflora* and *C. rubella* were defined as the out‐group to the rest of *Capsella* species (100 BS), following the topologies of Douglas et al. (2015), Guo et al. (2017), Hurka et al. (2012), Kryvokhyzha, Salcedo et al. (2019), and Walden et al. (2020). The analysis of the reduced dataset (i.e., dataset with accessions exhibiting <5% admixture) including only 57 accessions retrieved the same topology but with a better bootstrap support within *C. orientalis* (Figure S5). The following clades were inferred: *Capsella rubella* (100 BS), *C. grandiflora* (77 BS), Mediterranean *C. bursa‐pastoris* (100 BS), *C. thracica* (96 BS), Eurasian *C. bursa‐pastoris* (80 BS), South Kazakhstan *C. orientalis* (83 BS), Tabagartai–Dzhungarian Alatau *C. orientalis* (100 BS), Central Asian *C. orientalis* (100 BS), North Kazakhstan *C. orientalis* (73 BS), and Altai *C. orientalis* (98 BS). The only well‐supported sister group relationship was inferred between Altai and North Kazakhstan *C. orientalis* (100 BS). For easier depiction of the phylogenetic relationships within *Capsella*, a scheme based on the topology retrieved from the unreduced SVDQ analysis was reconstructed (Figure 3).

### 3.3 Time divergence estimation

All four time divergence analyses led to congruent results, despite the single secondary calibration points originating from different unrelated studies with different taxon sampling and genetic datasets. All parameters in the analyses converged to the stationary distribution (ESS > 200) and the overall topologies corresponded to the other phylogenetic studies (Douglas et al., 2015; Guo et al., 2017; Hurka et al., 2012; Kryvokhyzha, Salcedo et al., 2019; Walden et al., 2020). The crown age of *Capsella* was estimated to approximately 0.75 Ma, the split of *C. grandiflora* and *C. rubella* to approximately 0.19 Ma, and the split of *C. bursa‐pastoris*, *C. thracica,* and *C. orientalis* to 0.13 Ma (Figure 3). All three splits were placed into the Middle Pleistocene, mostly into MIS15, MIS7, and MIS6 (Figure 3). The crown age of *C. grandiflora* was dated at around 67 kya and the crown age of *C. rubella* at 26 kya, both placed into Late Pleistocene. The crown age of a clade consisting of *C. bursa‐pastoris* and *C. thracica* was estimated to the Middle Holocene at around 6.7 kya and the diversification of *C. orientalis* occurred in the Late Holocene approximately 2.1 kya (Figure 3).

### 3.4 Inference of genetic structure according to STRUCTURE

**FIGURE 4:**
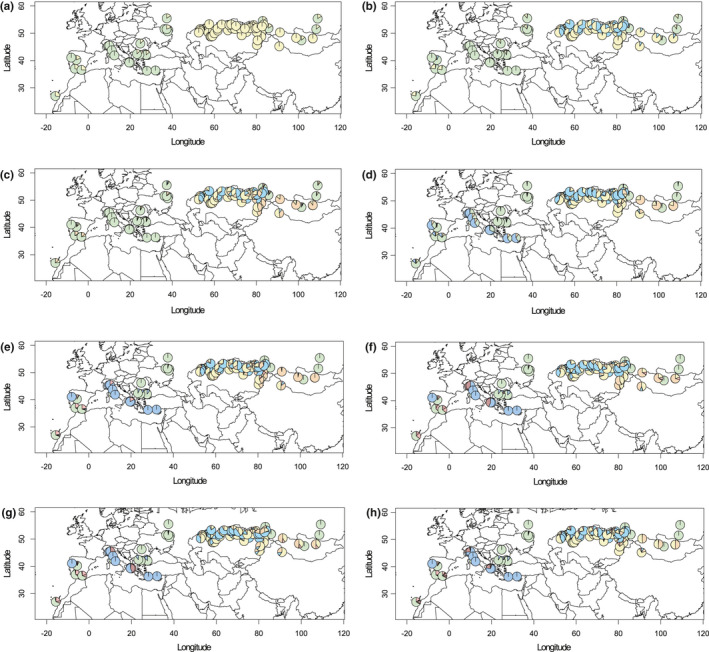
Genetic structure of sampled *Capsella* populations. Genetic cluster membership was inferred using STRUCTURE, showed as pie charts for *K* = 2 (a), *K* = 3 (b), *K* = 4 (c), *K* = 5 (d), *K* = 6 (e), *K* = 7 (f), *K* = 8 (g), and *K* = 9 (h)

**FIGURE 5:**
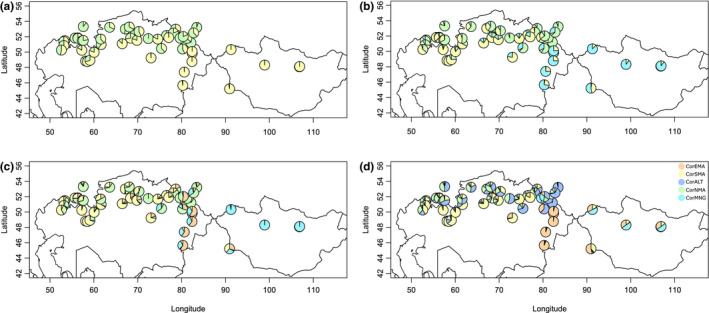
Geographic distribution of sampled *Capsella orientalis* populations. Genetic cluster membership was inferred using STRUCTURE, showed as pie charts for *K* = 2 (a), *K* = 3 (b), *K* = 4 (c), and *K* = 5 (d). Color coding as in the Files S4, S7, and S9, and is as follows: North Kazakhstan *C. orientalis* cluster in tea green (CorNMA), South Kazakhstan *C. orientalis* cluster in lemon yellow (CorSMA), Central Asian *C. orientalis* cluster in celeste (CorMNG), Tabagartai–Dzhungarian Alatau cluster *C. orientalis* cluster in deep champagne (CorEMA), and Altai *C. orientalis* cluster in baby blue (CorALT)

The highest Δ*K* value in the *Capsella* dataset was inferred for *K* = 6, while in the *Capsella orientalis* dataset the highest Δ*K* value was retrieved at *K* = 3 (Figure S6). The *Capsella* dataset subsequently splits in the following way: *K* = 2 separated *C. orientalis* from the rest, with *C. bursa‐pastoris* accessions exhibiting moderate introgression (Figure 4a). At *K* = 3 and *K* = 4, subsequent fragmentation within *C. orientalis* was observed, mirrored also in the LEA analysis in *Capsella* (see below and Figure S7) as well as in *C. orientalis* datasets (Figures S7 and S8). At *K* = 5, the Mediterranean diploid branch got separated, and at *K* = 6, the introgression of *C. grandiflora* was observed in the Mediterranean genetic cluster of *C. bursa‐pastoris* (Figure 4d,e). Subsequent clustering delimited Mediterranean *C. grandiflora* from genetically uniform *C. rubella* (Figure 4f). Further clustering could not be inferred from Figure 4 because it resulted in new minor genetic clusters embedded in *C. orientalis*. The *Capsella orientalis* dataset clustered in the following manner. At *K* = 2, northern and southern *C. orientalis* genetic clusters were inferred (Figure 5a). The southern cluster subsequently split up into a Tabagartai–Dzhungarian Alatau and Central Asian cluster, and South Kazakhstan cluster (at *K* = 3). The division ran across the southern foothills of the Altai Mountains, southwards to the Tian Shan Mountains along the Tabagartai–Dzhungarian Alatau mountain chain. At *K* = 4, a new genetic cluster mixed with the Central Asian genetic cluster was inferred along the Tabagartai–Dzhungarian Alatau mountain chain and at *K* = 5 an Altai genetic cluster emerged (Figure 5c,d). STRUCTURE genetic clustering did not strictly follow the *Capsella* species boundaries.

### 3.5 Inference of genetic structure according to LEA

The lowest cross‐entropy value in the *Capsella* dataset was inferred for *K* = 10 (albeit without a clear signal), while no optimal *K* value was inferred for *Capsella orientalis* dataset (Figure S6). The *Capsella* dataset subsequently split in the following way. The deepest split at *K* = 2 was not species specific. *Capsella bursa‐pastoris* and its sister species *C. thracica* clustered together, whereas *C. orientalis* clustered separately (Figure S7A). Sister species *C. grandiflora* and *C. rubella* were assigned to both clusters to approximately 50%. At *K* = 3, *C. grandiflora* and *C. rubella* were retrieved as a separate cluster (Figure S7B). At *K* = 4, *C. thracica* was retrieved as an individual species and at *K* = 5 the first intraspecific differentiation took place (Figure S7C,D). Further intraspecific split followed at *K* = 6, *K* = 7, and *K* = 8, inferring subpopulation structure within *C. orientalis* and splitting the *C. bursa‐pastoris* clade into a Mediterranean and Eurasian clade (Figure S7E,F,G). At *K* = 9, *C. grandiflora* was recognized as a separate cluster from *C. rubella* (Figure S7H), and at *K* = 10, the population genetic structure of *C. orientalis* reflected the same structuring as inferred at *K* = 5 from the *Capsella orientalis* dataset (Figure S8) and was as follows. At *K* = 2, a northern and southern *C. orientalis* cluster was inferred, which subsequently fragmented into a Central Asian cluster (at *K* = 3, Figure S8A), a Tabagartai–Dzhungarian Alatau cluster (at *K* = 4, Figure S8B), and an Altai cluster (at *K* = 5, Figure S8C). LEA genetic clustering mostly followed the *Capsella* species boundaries.

### 3.6 Inference of genetic structure according to DAPC

DAPC analysis of the *Capsella* dataset followed species‐specific genetic clustering. However, it failed to delimit *C. rubella* from *C. grandiflora* along all *K*‐values spanning from *K* = 3 to *K* = 9 (Figure S9). Close relationship between *C. bursa‐pastoris* and *C. thracica* was inferred from all *K*‐values. No extensive subclustering within *Capsella orientalis* was inferred (Figure S9). DAPC analysis of *Capsella orientalis* dataset retrieved the same substructure as inferred from LEA (Figures [Link], [Link]) and comparable to the one inferred from STRUCTURE (Figure 5). Recognized as separate clusters already at deeper nodes (inferred at *K* = 3 and *K* = 4 in the *Capsella orientalis* dataset, and at *K* = 7 and *K* = 9 in the *Capsella* dataset in LEA, respectively), the Tabagartai–Dzhungarian Alatau and Central Asian clusters were spatially well defined from the rest of *Capsella orientalis* populations. These were followed by the spatially still well separated Altai cluster, indicating a closer genetic relationship (Figure S10). The North Kazakhstan and South Kazakhstan *C. orientalis* clusters were still recognized as two, albeit partially overlapping, independent clusters (Figure S10).

### 3.7 Ecological niche modeling

**FIGURE 6:**
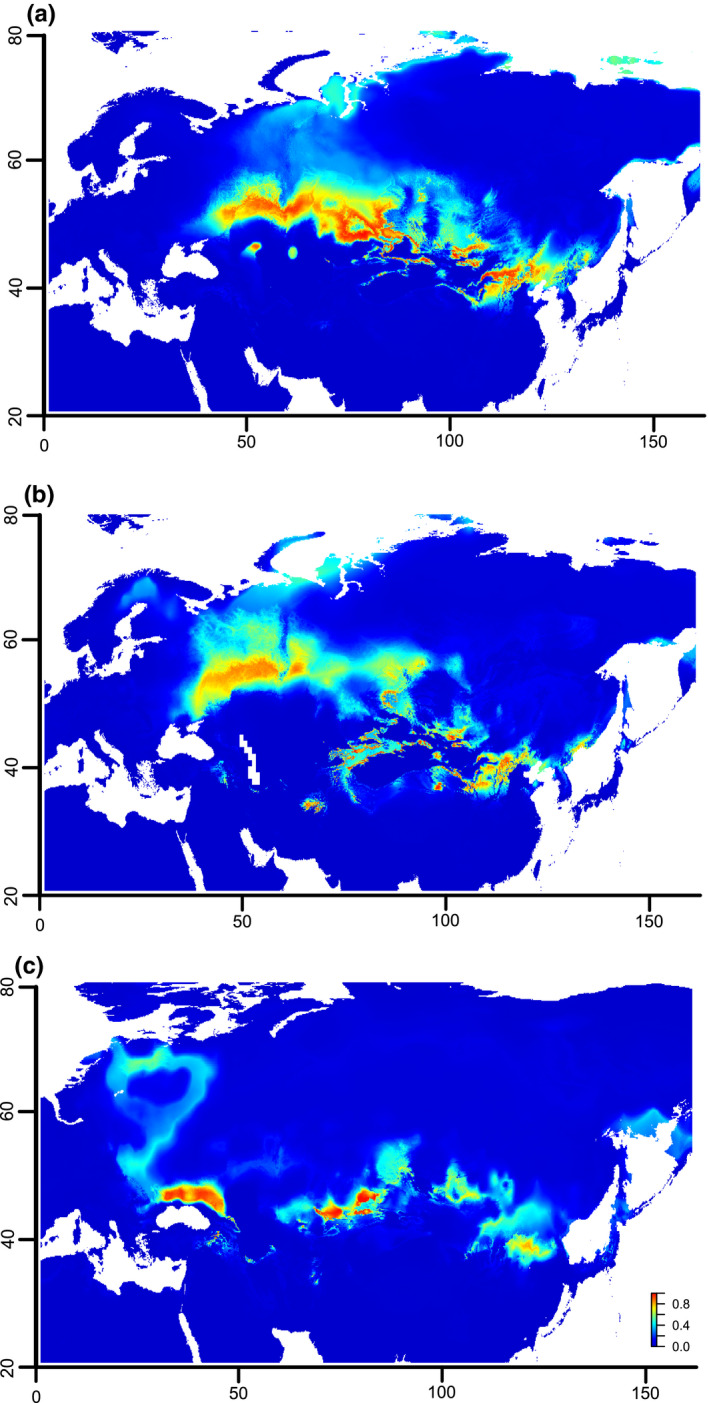
Predicted habitat suitability for *Capsella orientalis* during present time (a), Mid Holocene (b) and Last Glacial Maximum (c), respectively, as inferred from ecological niche modeling. Warmer colors represent areas of higher probability for the species’ presence, while colder colors represent areas with lower probability for its presence. Note that the analysis retrieves only the habitat suitability, but not necessarily the realized ecological niche of *C. orientalis*

The best model (LQH 0.5; AUC = 0.8326) inferred from the ENMeval appropriately encompassed the current known distribution area of *Capsella orientalis* (Figure 6a). The maximum entropy algorithm indicated that the climatically suitable habitats were gradually reduced toward the east and west throughout the Holocene into the south Central Asian Mountain Chains and northern Pontic steppe (Figure 6b). During the LGM, the only climatically suitable habitats according to our niche modeling were the Black Sea coast in the west and South and South‐East Kazakhstan in the east, with partially climatically suitable habitats also in Eastern China (Figure 6c).

### 3.8 Population genetics analysis of *Capsella orientalis*

No extensive correlation of geography either with the molecular diversity indexes or with the number of private alleles were inferred (Figure S11). Individual populations with the highest genetic diversity indices (Θ_S_ and Θ_π_) were from Altai, South Kazakhstan, and North Kazakhstan. Individual populations with the lowest genetic diversity according to the Θ_S_ and Θ_π_ indices were in Altai and Mongolia but also in North Kazakhstan and Tabagartai/Dzhungarian Alatau (Figure S12–1). The highest number of substitution sites per gene copy were found in Altai populations, North Kazakhstan, and South Kazakhstan, and the lowest number of substitution sites per gene copy were recorded in Mongolian populations, Altai, and North Kazakhstan (Figure S12–1). The highest number of private substitution sites per gene copy, however, was found in South Kazakhstan populations and in two Altai populations. Lowest number of private substitution sites per gene copy were recovered from North Kazakhstan, Altai, and Central Asian populations (Figure S12–1). When populations were pooled together according to geography, the highest genetic diversity in terms of Θ_S_, Θ_π_, and proportion of substitution sites per gene copy and private substitution sites per gene copy was retrieved from the South Kazakhstan cluster, and the lowest genetic diversity was inferred from the Mongolia genetic cluster (Figure S12–2).

*F*‐statistics uncovered that the most genetically similar populations are in North Kazakhstan with values between 0.08 and 0.09. Contrarily, the highest *F*_ST_ values (0.60 > *F*_ST_ > 0.40) were inferred between some Central Asian, Altai, and South Kazakhstan populations (Figure S12–3). When pooled together according to geography, F_ST_ analysis indicated that the most similar genetic clusters were the North Kazakhstan and South Kazakhstan genetic clusters (*F*_ST_ = 0.039). Genetically, the most divergent genetic cluster was the Tabagartai/Dzhungarian cluster that differed the most from the Altai (*F*_ST_ = 0.115) and Central Asian genetic cluster (*F*_ST_ = 0.110; Figure S12–4).

AMOVA uncovered that the genetic divergence among the five genetic clusters was low at only about 3.70% (Figure S12–5). 10.60% of genetic diversity could be explained among populations, and the highest percentage of genetic variation could be explained along individuals within populations at 81.83%. The average observed heterozygosity H_O_ was at 0.071 near zero, and the average expected heterozygosity *H*_E_ was 0.489 (Figure S12–5). The degree of inbreeding within populations (*F*_IS_) was high at 0.955. Inbreeding coefficient of an individual relative to the total population (*F*_IT_) was also high at 0.961. The variance among subpopulations within groups (*F*_SC_) was close to zero at 0.110 as it was the variance among groups relative to the total variance (*F*_CT_) at 0.037 (Figure S12–5). All AMOVA results were highly significant with *p* < .005.

## 4 DISCUSSION

### 4.1 Dated phylogeny of evolutionary lineages within *Capsella*

**FIGURE 7:**
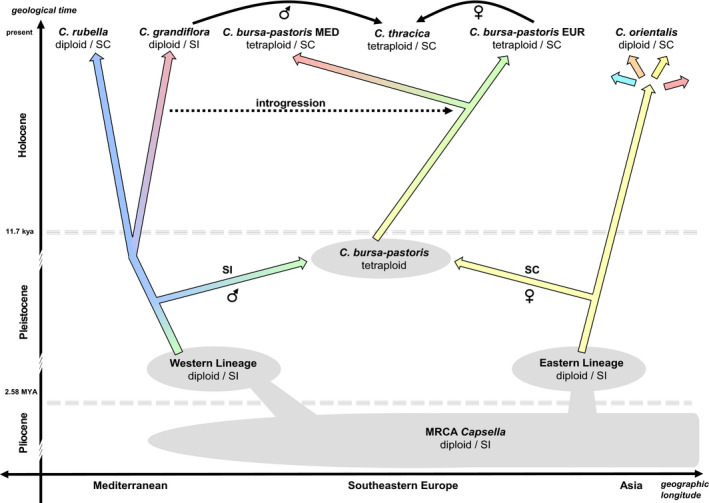
Outline of the evolutionary history of the genus *Capsella* placed into space and time. The color coding of the arrows corresponds to the color coding in Figure 4. Note that the geological time axis as well as the geographical latitude axis are not to scale

Our SNP‐based data clearly confirm the five species concept for *Capsella* (Chater, 1964; Hurka et al., 2012) and corroborate the identification of two ancestral evolutionary lineages within the genus as already outlined in Hurka et al. (2012): *Capsella grandiflora* and *C. rubella* on the Mediterranean side, and *C. orientalis* on the Eurasian side. The two allopolyploids *C. bursa‐pastoris* and *C. thracica* are geographically and genetically placed in between, albeit the geographic signal of the former one is obscured due to its immense colonization potential (Figures 3 and 7). Our SNP data also support the two lineages within *C. bursa‐pastoris* as previously revealed by multilocus isozyme analyses—a Mediterranean and a temperate Eurasian lineage (Wesse et al., 2021)—and are in agreement with the SNP‐based analyses of Cornille et al. (2016). These authors detected a European/Russian and a “Middle Eastern” (which we refer to as “Mediterranean”) *C. bursa‐pastoris* genetic cluster in the geographic area covered by our study. The origin of *C. thracica* is within the temperate *C. bursa‐pastoris* lineage, matching the geographical distribution areas of the two taxa.

The main conclusion from hitherto published *Capsella*‐dating analyses and our current analysis is that the tribe Camelineae originated in the second half of the Miocene and the stem age of *Capsella* is of Pliocene age (Figures 3 and 7, Figure S13). During the Pleistocene, *Capsella* split into two basal lineages, an eastern and a western lineage, and subsequent splits within the two lineages occurred during the Middle to Late Pleistocene and in postglacial times (Figures 3 and 7).

### 4.2 Phylogeography of *Capsella*

#### 4.2.1 The genus *Capsella*

It has already been hypothesized that the distribution area of the most recent common ancestor (MRCA) of *Capsella* stretched across Eastern Europe and/or western Siberia (Hurka et al., 2012). Based on the present results, we can be more precise. The sister genus *Catolobus* (Couvreur et al., 2010; Huang et al., 2016) is found in temperate climate zones of Eurasia stretching from Japan, Russian Far East, and China to southeastern Europe, and inhabits woodlands, grasslands, and riverbanks often as a ruderal weed. *Catolobus* thus shares ecogeographical characters and distribution area with *Capsella*. Morphological evidence (indumentum, trichome types, branching patterns) and ploidy level (2n = 2x = 16 for the two basal branching lineages in *Capsella*) also support the close relationship.

We assume the biogeographical roots of the genus *Capsella* in Middle Asia, in the area including the northern Caspian Sea coast region and Kazakhstan (Western Asiatic subregion of the Irano‐Turanian floristic region sensu Takhtajan, 1986, regional subcenter IT3 sensu Manafzadeh et al., 2017). This is suggested by the present distribution area of *C. orientalis* (Figure 2). During the Pliocene, the MRCA of *Capsella* extended its range out of Asia and westwards into southeastern Europe and eventually even further west into the Mediterranean region. This timing corresponds with the estimated stem age of *Capsella* (split between *Catolobus* and *Capsella*) of ca. 4 ± 2 Ma (Žerdoner Čalasan et al., 2019). Theoretically, there are two possible east‐to‐west migration routes, (a) a “southern route” south of the Caspian and Black Sea to the eastern Mediterranean region and from there to southeastern Europe, and (b) a “northern route” along the steppe biome to southeastern Europe and perhaps even further west into the Mediterranean climate zone. Many arguments are in favor of the northern route whereas the southern route appears very unlikely as explained below.

The "southern route” scenario: Mediterranean steppes are thought to be descendants of the Anatolian steppes that expanded throughout the Mediterranean during the Pliocene (Suc et al., 2018). This route, however, is discussed for xeric steppe elements only (Jia & Bartish, 2018; Lauterbach et al., 2019; Manafzadeh et al., 2014; Žerdoner Čalasan et al., 2021), and *Capsella* does not belong to this ecological group. The main argument against an Early Pliocene southern *Capsella* route is the estimated divergence time between the two basally branching lineages, the Mediterranean *C. grandiflora/rubella* and the continental *C. orientalis* lineage of ca. 1 Ma or less, clearly indicating a Pleistocene and not a Pliocene origin. A possible Late Pliocene/Early Pleistocene “southern route” did not exist because of the Akhchagyl and Apsheron transgressions of the Caspian Sea, which were continuously blocking access to the southern route from the north for a period of ca. 2 million years (from ca. 3 to 1 Ma: Popov et al., 2006; Svitoch, 2016). Later, the great deserts of southern Kazakhstan and Turkmenistan prevented migration from northern parts of Kazakhstan to the southwest (southern route). Thus, the scenario of ancestral *Capsella* reaching Europe via a “southern route” is highly improbable.

Instead, there is convincing evidence for the "northern route.” The spread of steppe elements from Asia to the Black Sea region since the Pliocene is clearly indicated by pollen records (e.g., Feurdean & Vasiliev, 2019; Popescu et al., 2010; Popova et al., 2017; Tarasov et al., 2000), and the climate/landscape history of the Eurasian steppe belt throughout the Neogene and Quaternary has been intensively studied (Grichuk (1989) and Velichko et al. (2005) for East European Plain; Arkhipov et al. (2005) for West Siberian Plain; Akhmetiev et al. (2005) for Kazakhstan Plains; see also Hurka et al. (2019) and references therein). These studies elucidate the importance of Euro‐Siberian steppe as a corridor for *Capsella* migration routes, which is mirrored in the present distribution area of *Capsella orientalis* (Figure 2).

In the Middle Pleistocene, the two diploid basal *Capsella* lineages evolved (Figures 3 and 7). Present distribution area and climate envelope identify *C. orientalis* and *C. grandiflora*/*rubella* as true steppe and Mediterranean elements, respectively. Their Pleistocene biogeography was heavily affected by the numerous cold‐warm macrocycles of the Pleistocene. On the East European Plain, extreme changes of landscapes occurred during the last 800 kya (Middle to Late Pleistocene: Don, Oka, Dnieper, Valdai glaciations and corresponding interglacial warm phases). Severity and duration of cold phases increased and development of permafrost, formation of large continental ice sheets, and loess deposition intensified. The Don glaciation was the greatest glaciation on the East European Plain (correlated with the “Cromer Complex” Stage in NW Europe: Velichko et al., 2011a). Periglacial cold steppe spread over the East European Plain north of the Black and Caspian Sea. Interglacial periods were characterized by temperate steppes, forest‐steppe, and forests (Grichuk, 1989, 1992a; Velichko et al., 2005). Contrarily, the Mediterranean region including the Balkan Peninsula was not affected as much. Mediterranean steppes thus continued to exist during the Pleistocene, but around 1 Ma temperature gradients became more important than humidity gradients (turnover from xeric to more thermic determinism sensu Suc et al., 2018).

#### 4.2.2 Capsella orientalis

The distribution map presented in Figure 2 comprises all recorded localities of *C. orientalis* known to us (literature surveys, herbarium studies, and own field collections). The distribution area stretches nearly 5,000 km from Eastern Europe (lower Don) to Central Asia (Mongolia) more or less along the Eurasian steppe belt. Its main distribution is in Kazakhstan.

Present differentiation within *C. orientalis* is of postglacial age and thus very young, which is also expressed by invariable ITS, ETS, and cpDNA sequences (data not shown). The *C. orientalis* lineage itself is of Middle Pleistocene age (Figure 3). Palaeoclimate modeling indicates that *C. orientalis* could survive the LGM in southern Kazakhstan and southeastern Europe (Figure 6). With the beginning of the Holocene, *C. orientalis* extended its range from the Asian refuge area northwards and eastwards into the Altai/Sayan and Tabagartai/Dzhungarian mountain chains and into Central Asia (Figure 5). This scenario is supported by the high number of private alleles and molecular diversity indexes Θ_S_ and Θ_π_, which on average are higher in southern Kazakhstan than in the recently colonized regions. Lowest genetic diversity was observed in Mongolia. However, discontinuous mosaic variation patterns were also observed (Figure 5; Figures [Link], [Link]). This overall genetic variation pattern is typical for recent colonization events (for *C. bursa‐pastoris* see for instance Hurka, 1984, 1990, Wesse et al., 2021). Interestingly, flowering behavior does not follow this pattern. *Capsella orientalis* flowers in the spring in the lowland steppe and forest‐steppe biomes of the Euro‐Siberian steppe belt ("planta vernalis,” Klokov, 1926) but significantly later in the mountain steppes of the Altai/Sayan Mountain Country and Mongolia (estimated by comparisons of collecting dates, data not shown). Flowering ecology of *C. orientalis* thus varies greatly with the environments and growing seasons. We conclude that the variation pattern at the molecular level reflects population histories, colonization processes like gene flow events, and genetic drift effects whereas variation at the phenotypic level reflects adaption to local environments (including the altitude).

Population genetic data are in accordance with a predominantly selfing breeding system effective in natural populations (*H*_O_ = 0.0071, *H*_E_ = 0.489; *F*_IS_ = 0.955, *F*_IT_ = 0.961; see chapter Results) and confirm conclusions drawn from previous research (Bachmann et al., 2019; Hurka et al., 2012; Woźniak et al., 2020). Assuming that self‐compatibility (SC) is a derived character state (Durand et al., 2020), the question arises about the timing of the loss of self‐incompatibility (SI). Bachmann et al. (2019) argue for a loss of SI in *C. orientalis* between 2.6 Ma and 70 kya but closer to the upper bound, and provide evidence that *C. orientalis* was SC when it contributed to the origin of *C. bursa‐pastoris* at about 130 kya.

#### 4.2.3 *Capsella bursa*‐*pastoris*

It is evident from the STRUCTURE analysis (Figure 4) that *Capsella bursa‐pastoris* retained SNP sequences from both ancestral lineages. This argues for a hybrid origin of *C. bursa‐pastoris* between the two lineages, which has been confirmed by other genomic studies that show two subgenomes in *C. bursa‐pastoris*, one originating from the western and one from the eastern lineage (Douglas et al., 2015; Han et al., 2015; Kryvokhyzha, Salcedo et al., 2019). Furthermore, subsequent evidence argues that the maternal parent came from the eastern lineage represented by *Capsella orientalis* (Douglas et al., 2015; Hurka et al., 2012; Omelchenko et al., 2020). To interpret the polyploidization event in a phylogeographic framework, we have to postulate firstly, a split of the continuous MRCA distribution area of *Capsella* dividing it into two separated areas, and secondly, subsequent expansion out of the two part areas leading to a contact zone where the tetraploid ancestor of *C. bursa‐pastoris* arose through allopolyploidy.

Arguments for a MRCA range split during the Middle and Late Pleistocene as suggested by our time divergence estimation (Figure 3) are numerous and outlined above (see Discussion on the genus *Capsella*). During the cold stages, the interglacial steppe belt was replaced by periglacial cold steppe and tundra‐steppe north of the Black Sea. In the following interglacial, steppe and forest‐steppe expanded again in east‐to‐west direction. This eastward contraction and westward expansion of the steppe belt occurred several times during the climatic macrocycles (Grichuk, 1989; Velichko et al., 2005). In Asia, the steppe could move to the south whereas in southeastern Europe, the steppe could not migrate to the south and the continuous range of the MRCA was disrupted due to geophysical barriers (Black Sea and Caucasus). Its western partial distribution areal was pushed west of the Black Sea into the Balkan Peninsula and into the Mediterranean region. Westward range extensions of Pontic‐South Siberian steppe and forest‐steppe geoelements into the Balkan Peninsula are numerous (Horvat et al., 1974). The Thracian Plain connecting the southwestern East European Plain and Anatolia with the Mediterranean region is a well‐known gateway for plant migration in and out of the Balkan Peninsula (Horvat et al., 1974; Magyari et al., 2008; Suc et al., 2018; Turrill, 1929), and might have also facilitated the westward migration of *Capsella* into the Balkan Peninsula.

According to our divergence time estimations (Figure 3), the hybridization event leading to *C. bursa‐pastoris* took place during the last Pleistocene transition from glacial to interglacial, about 120,000 years ago, corresponding approximately to the last interglacial (LIG) and MIS 5e (Eem or Mikulino, respectively). During the climatic optimum of the LIG (about 120,000 years ago), nemoral forest grew around the Black Sea and northern parts of the Balkan Peninsula down to 40°N. It thus appears that the steppe belt was interrupted not only in glacial but also in interglacial times, and one might question a secondary contact zone. However, the nemoral forests mirror the condition only during climatic/vegetation optima of the interglacials. Outside of these optima, longer periods with open vegetation in form of grasslands or forest‐steppe existed (Leroy et al., 2011), and this opened up a short window for steppe plants to migrate out of their glacial refugia (western Balkan and eastern Black Sea/Kazakhstan), resulting in a secondary contact.

Predicted distribution of *C. grandiflora* at present, LGM and LIG based on ecological niche modeling support our assumption of range extension from Balkan Peninsula refugium toward the east into the Black Sea region (Han et al., 2015). We hypothesize that the gene pools of the two diploid basal lineages, which had been separated since the Middle Pleistocene, had already differentiated into the *C. grandiflora* and *C. orientalis* lineages before coming into secondary contact in the northern Black Sea coast region. In the secondary contact zone, *C. bursa‐pastoris* originated via hybridization and whole‐genome duplication (allotetraploidy). This was not a single event and must have taken place several times. Otherwise, the polymorphism within *C. bursa‐pastoris* as inferred from isozymes cannot be explained (Hurka et al., 2012). Douglas et al. (2015) also “clearly rule out a single polyploid origin from two haploid gametes”. Recently uncovered spontaneous somatic tetraploidization following hybridization between *C. orientalis* and *C. grandiflora* experimentally supports the origin of *C. bursa‐pastoris* (Bachmann et al., 2021) and offers further interpretation on the polyplodization process.

Genome duplications and their consequences as drivers of major evolutionary innovations are in focus of current research at the genomic level (Nieto Feliner et al., 2020; Soltis & Soltis, 2020; Spoelhof et al., 2020; Walden et al., 2020). In the recent years, allopolyploid *C. bursa‐pastoris* received special attention because of its young age (Douglas et al., 2015; Kasianov et al., 2017; Kryvokhyzha, Salcedo et al., 2019, Kryvokhyzha, Milesi et al., 2019). The genome duplication in *C. bursa‐pastoris* coincides with its enormous colonization ability to migrate into different climate zones and colonize regions far above those inhabited by its parent species (Hurka & Neuffer, 1997; Wesse et al., 2021). It would appear that *C. bursa‐pastoris* quickly spread over Eurasia shortly after its origin in the Late Pleistocene. This is supported by fossil seeds of *C. bursa‐pastoris* reported from interglacial deposits at Ilford, Essex, England (West et al., 1964). The fossil‐bearing sediments correlate with MIS 5e (Eemian of continental Europe). Distribution of *C. bursa‐pastoris* on the British Isles at the LIG was predicted by palaeomodelling as well (Han et al., 2015) and thus supports the assumption of quick and early range extension after its origin in the southwestern East European Plain.

#### 4.2.4 *Capsella bursa*‐*pastoris*, MED, Mediterranean lineage

As outlined above, two intraspecific lineages are recognized in *C. bursa‐pastoris*, one predominantly in Mediterranean‐like climate regions (*C. bursa‐pastoris* MED) and the other in more temperate climate regions (*C. bursa‐pastoris* EUR). These two lineages are of Holocene age (Figure 3). We hypothesize that the Mediterranean *C. bursa‐pastoris* had contact with the diploid Mediterranean species *C. grandiflora/rubella* and that the MED lineage originated from introgression of *C. grandiflora/rubella* gene material into the *C. bursa‐pastoris* genome (Figure 4e). This gene flow may have supported the adaptation to Mediterranean climates during the postglacial colonization. Distribution models of the MED and EUR lineages are clearly in favor of respective climate preferences (Wesse et al., 2021). The observed worldwide distribution patterns of genetic variation in the range of *C. bursa‐pastoris* are the outcome of both migration/colonization history and environmental filtering due to climate preadaptation.

Mixed populations of diploid *C. grandiflora/rubella* and tetraploid *C. bursa‐pastoris* are common in the Mediterranean region. These species often hybridize, and the triploid hybrid, known under the name *C. gracilis* Gren., can produce flowers. The hybrid is mostly sterile but fertile intermediates sometimes occur (reported by Chater, 1993). Thus, this triploid stage might eventually served as a bridge for introgression of diploid gene material across ploidy barriers into *C. bursa‐pastoris*. Slotte et al. (2008) record introgression of *C. rubella* into *C. bursa‐pastoris,* and adaptive introgression from the diploid congeners into *C. bursa‐pastoris* has been demonstrated before as well by Han et al. (2015).

#### 4.2.5 Capsella thracica

In the Early Holocene, *C. thracica* originated by hybridization between the Eurasian lineage of *C. bursa‐pastoris* and *C. grandiflora* in southwestern Europe. This is clearly confirmed by our time divergence estimation (Figure 3) and by the genetic structure analysis (Figure 4). During the LGM (20,000–18,000 years), periglacial steppes covered the landscape from the northern Black Sea coast to the Altai‐Tian Shan‐Pamir mountain systems (Grichuk, 1992a, 1992b; Velichko & Isayeva, 1992) and divided the preceding Last Interglacial (LIG) grassland/dry shrubland/nemoral forest belt into a Mediterranean and Asian area, which became closed again in the Holocene. This in turn opened up again migration routes for steppe and Mediterranean elements. A strong Mediterranean influence on the western Pontic steppe vegetation has been observed before (Magyari et al., 2008; Turrill, 1929). The origin of the sub‐Mediterranean geoelements in the steppe vegetation has long been discussed, and it is hypothesized that many of them invaded the region during the Holocene from their Pleistocene refuge areas in the Balkan Peninsula (Tzonev et al., 2006 and references therein).

The evolutionary history of *C. grandiflora* fits well into this picture. This species is a typical Mediterranean element whose distribution area was drastically reduced during the glaciation periods. We assume that during the Early Holocene *C. grandiflora* extended its range from its LGM Balkan refuge to the east into the Thracian Plain similarly to the possible pre‐LIG expansion (Han et al., 2015). In the Thracian Plain, the hybridization occurred analogously to the one resulting in the origin of *C. bursa‐pastoris* as supported by the two ITS copies, one from *C. bursa‐pastoris* and one from *C. grandiflora* (Hurka et al., 2012). In agreement with these findings are our SNP data. STRUCTURE analysis retrieved a signal of the Mediterranean diploid *Capsella* species from all the investigated *Capsella thracica* populations (Figure 4). This Mediterranean SNP signal is with all probability from *C. grandiflora* since no *C. rubella* ITS sequence signal was ever inferred in *C. thracica*. Due to continuous backcrossing of *C. thracica* with its maternal EUR *Capsella bursa‐pastoris*, the proportion of the Mediterranean signal is minor and might even eventually disappear.

## CONFLICT OF INTEREST

The authors declare no conflict of interest.

## AUTHOR CONTRIBUTION

**Anže Žerdoner Čalasan:** Data curation (lead); Formal analysis (lead); Investigation (lead); Methodology (lead); Visualization (lead); Writing‐original draft (lead); Writing‐review & editing (lead). **Herbert Hurka:** Conceptualization (lead); Funding acquisition (lead); Supervision (lead); Writing‐original draft (equal); Writing‐review & editing (equal). **Dmitry A. German:** Data curation (supporting); Investigation (supporting); Writing‐original draft (supporting); Writing‐review & editing (supporting). **Simon Pfanzelt:** Data curation (supporting); Investigation (supporting); Writing‐original draft (supporting); Writing‐review & editing (supporting). **Frank R. Blattner:** Funding acquisition (equal); Writing‐original draft (supporting); Writing‐review & editing (supporting). **Anna Seidl:** Data curation (supporting); Writing‐original draft (supporting); Writing‐review & editing (supporting). **Barbara Neuffer:** Funding acquisition (equal); Supervision (equal); Writing‐original draft (equal); Writing‐review & editing (equal).

## Supporting information

File S1

File S2

File S3

File S4

File S5

File S6

File S7

File S8

File S9

File S10

File S11

File S12

File S13

## Data Availability

Data Accessibility: All the ipyrad output files of the “ori” as well as “caps” datasets are available on Dryad under https://doi.org/10.5061/dryad.j6q573nf3.
