# Supplementary material for: Pleistocene dynamics of the Eurasian steppe as a driving force of evolution: Phylogenetic history of the genus Capsella (Brassicaceae)
Source: Ecol Evol. 2021 Aug 18;11(18):12697–713. doi: 10.1002/ece3.8015 (PMC8462161; doi:10.1002/ece3.8015)
Supplement: Supplementary file 1 — File S1 [file ECE3-11-12697-s012.docx]

**Supplementary File 1: A complete taxon sample** of different *Capsella* species, analysed with GBS. Note that the three repetitions for internal assessment of the protocol are labelled with ‘a’ and ‘b’ following the population label, respectively. Abbreviations: Nr. number, C Central, E East, S South, SE South-East, SW South-West, W West.

| **Species** | **Voucher Nr.** | **Population Nr.** | **Individuum Nr.** | **Latitude** | **Longitude** | **Region** |
| --- | --- | --- | --- | --- | --- | --- |
| *Capsella bursa-pastoris* | OSBU1229 | CBP1229 | A160cbp1229_18 | 40.21 | -5.60 | Mediterranean |
| *Capsella bursa-pastoris* | OSBU1272 | CBP1272 | A159cbp1272_7 | 37.00 | -6.33 | Mediterranean |
| *Capsella bursa-pastoris* | OSBU1282 | CBP1282 | A158cbp1282_9 | 36.57 | -3.20 | Mediterranean |
| *Capsella bursa-pastoris* | OSBU1455 | CBP1455 | A153cbp1455_2 | 42.53 | 11.41 | Italy |
| *Capsella bursa-pastoris* | OSBU1942 | CBP1942 | A154cbp1942_5 | 51.23 | 38.08 | SW_Russia |
| *Capsella bursa-pastoris* | OSBU1942 | CBP1942 | A155cbp1942_1 | 51.23 | 38.08 | SW_Russia |
| *Capsella bursa-pastoris* | OSBU2044 | CBP2044 | A162cbp2044_2 | 55.48 | 37.38 | SW_Russia |
| *Capsella bursa-pastoris* | OSBU2046 | CBP2046 | A163cbp2046_1 | 51.50 | 108.52 | SE_Russia |
| *Capsella bursa-pastoris* | OSBU2048 | CBP2048 | A164cbp2048_1 | 55.37 | 109.19 | SE_Russia |
| *Capsella bursa-pastoris* | OSBU2050 | CBP2050 | A165cbp2050_3 | 54.50 | 83.06 | Altai |
| *Capsella bursa-pastoris* | OSBU2222 | CBP2222 | A166cbp2222_2 | 51.86 | 85.83 | Altai |
| *Capsella bursa-pastoris* | OSBU2244 | CBP2244 | A040cbp2244_1 | 27.00 | -15.62 | Mediterranean |
| *Capsella bursa-pastoris* | OSBU2361 | CBP2361 | A161cbp2361_1 | 46.22 | 24.79 | Romania |
| *Capsella bursa-pastoris* | OSBU26113 | CBP26113 | A142cbp26113_3 | 47.43 | 101.48 | Mongolia |
| *Capsella bursa-pastoris* | OSBU26113 | CBP26113 | A143cbp26113_8 | 47.43 | 101.48 | Mongolia |
| *Capsella bursa-pastoris* | OSBU26113 | CBP26113 | A144cbp26113_2 | 47.43 | 101.48 | Mongolia |
| *Capsella bursa-pastoris* | OSBU26113 | CBP26113 | A145cbp26113_5 | 47.43 | 101.48 | Mongolia |
| *Capsella bursa-pastoris* | OSBU26113 | CBP26113 | A146cbp26113_7 | 47.43 | 101.48 | Mongolia |
| *Capsella bursa-pastoris* | OSBU27014 | CBP27014 | A157cbp27014_1 | 51.54 | 36.31 | SW_Russia |
| *Capsella bursa-pastoris* | OSBU27100 | CBP27100 | A152cbp27100_1 | 50.68 | 37.81 | SW_Russia |
| *Capsella bursa-pastoris* | OSBU27136 | CBP27136 | A167cbp27136_1 | 51.50 | 37.30 | SW_Russia |
| *Capsella bursa-pastoris* | OSBU27189 | CBP27189 | A151cbp27189_2 | 51.42 | 83.55 | Altai |
| *Capsella bursa-pastoris* | OSBU27493 | CBP27493 | A156cbp27493_3 | 51.83 | 85.79 | Altai |
| *Capsella grandiflora* | OSBU1840 | CG1840 | A019cg1840_4 | 39.46 | 19.52 | Greece |
| *Capsella grandiflora* | OSBU1857 | CG1857 | A021cg1857_2 | 39.23 | 20.03 | Greece |
| *Capsella grandiflora* | OSBU1861 | CG1861 | A015cg1861_11 | 39.28 | 19.53 | Greece |
| *Capsella grandiflora* | OSBU2015 | CG2015 | A001cg2015_1 | 45.30 | 9.52 | Italy |
| *Capsella grandiflora* | OSBU2016 | CG2016 | A004cg2016_1 | 45.34 | 9.51 | Italy |
| *Capsella grandiflora* | OSBU2019 | CG2019 | A008cg2019_2 | 45.39 | 10.04 | Italy |
| *Capsella grandiflora* | OSBU2037 | CG2037 | A011cg2037_2 | 45.46 | 10.06 | Italy |
| *Capsella orientalis* | OSBU1933 | CO1933 | A069co1933_3 | 50.33 | 91.26 | Mongolia |
| *Capsella orientalis* | OSBU1933 | CO1933 | A070co1933_6 | 50.33 | 91.26 | Mongolia |
| *Capsella orientalis* | OSBU1933 | CO1933 | A071co1933_4 | 50.33 | 91.26 | Mongolia |
| *Capsella orientalis* | OSBU1933 | CO1933 | A072co1933_2 | 50.33 | 91.26 | Mongolia |
| *Capsella orientalis* | OSBU1933 | CO1933 | A073co1933_5 | 50.33 | 91.26 | Mongolia |
| *Capsella orientalis* | OSBU1939 | CO1939 | A189co1939_1 | 52.16 | 76.57 | C_Kazakhstan |
| *Capsella orientalis* | OSBU1940 | CO1940 | A190co1940_2 | 50.47 | 75.41 | C_Kazakhstan |
| *Capsella orientalis* | OSBU1978 | CO1978 | A084co1978_6 | 51.06 | 81.54 | Altai |
| *Capsella orientalis* | OSBU1979 | CO1979 | A066co1979_6 | 51.07 | 81.48 | Altai |
| *Capsella orientalis* | OSBU1979 | CO1979 | A067co1979_2 | 51.07 | 81.48 | Altai |
| *Capsella orientalis* | OSBU1979 | CO1979 | A068co1979_1 | 51.07 | 81.48 | Altai |
| *Capsella orientalis* | OSBU1980 | CO1980 | A061co1980_1 | 51.10 | 81.40 | Altai |
| *Capsella orientalis* | OSBU1980 | CO1980 | A062co1980_4 | 51.10 | 81.40 | Altai |
| *Capsella orientalis* | OSBU1980 | CO1980 | A063co1980_5 | 51.10 | 81.40 | Altai |
| *Capsella orientalis* | OSBU1980 | CO1980 | A064co1980_3 | 51.10 | 81.40 | Altai |
| *Capsella orientalis* | OSBU1980 | CO1980 | A065co1980_2 | 51.10 | 81.40 | Altai |
| *Capsella orientalis* | OSBU1981 | CO1981 | A264co1981_5 | 51.08 | 81.36 | Altai |
| *Capsella orientalis* | OSBU1981 | CO1981 | A265co1981_3 | 51.08 | 81.36 | Altai |
| *Capsella orientalis* | OSBU1981 | CO1981 | A266co1981_6 | 51.08 | 81.36 | Altai |
| *Capsella orientalis* | OSBU1981 | CO1981 | A267co1981_4 | 51.08 | 81.36 | Altai |
| *Capsella orientalis* | OSBU1981 | CO1981 | A268co1981_1 | 51.08 | 81.36 | Altai |
| *Capsella orientalis* | OSBU1982 | CO1982 | A269co1982_3 | 51.30 | 81.13 | Altai |
| *Capsella orientalis* | OSBU1982 | CO1982 | A270co1982_2 | 51.30 | 81.13 | Altai |
| *Capsella orientalis* | OSBU1982 | CO1982 | A271co1982_6 | 51.30 | 81.13 | Altai |
| *Capsella orientalis* | OSBU1982 | CO1982 | A272co1982_1 | 51.30 | 81.13 | Altai |
| *Capsella orientalis* | OSBU1982 | CO1982 | A273co1982_4 | 51.30 | 81.13 | Altai |
| *Capsella orientalis* | OSBU1983 | CO1983 | A274co1983_6 | 51.22 | 82.12 | Altai |
| *Capsella orientalis* | OSBU1983 | CO1983 | A275co1983_2 | 51.22 | 82.12 | Altai |
| *Capsella orientalis* | OSBU1983 | CO1983 | A276co1983_1 | 51.22 | 82.12 | Altai |
| *Capsella orientalis* | OSBU1983 | CO1983 | A277co1983_3 | 51.22 | 82.12 | Altai |
| *Capsella orientalis* | OSBU1983 | CO1983 | A278co1983_4 | 51.22 | 82.12 | Altai |
| *Capsella orientalis* | OSBU1984 | CO1984 | A279co1984_4 | 53.21 | 83.44 | Altai |
| *Capsella orientalis* | OSBU1984 | CO1984 | A280co1984_2 | 53.21 | 83.44 | Altai |
| *Capsella orientalis* | OSBU1984 | CO1984 | A281co1984_6 | 53.21 | 83.44 | Altai |
| *Capsella orientalis* | OSBU1985 | CO1985 | A101co1985_1 | 53.21 | 83.44 | Altai |
| *Capsella orientalis* | OSBU1985 | CO1985 | A102co1985_2 | 53.21 | 83.44 | Altai |
| *Capsella orientalis* | OSBU1985 | CO1985 | A103co1985_3 | 53.21 | 83.44 | Altai |
| *Capsella orientalis* | OSBU1985 | CO1985 | A104co1985_6 | 53.21 | 83.44 | Altai |
| *Capsella orientalis* | OSBU1985 | CO1985 | A105co1985_4 | 53.21 | 83.44 | Altai |
| *Capsella orientalis* | OSBU2223 | CO2223 | A096co2223_2 | 45.22 | 90.93 | Mongolia |
| *Capsella orientalis* | OSBU2223 | CO2223 | A097co2223_1 | 45.22 | 90.93 | Mongolia |
| *Capsella orientalis* | OSBU2223 | CO2223 | A098co2223_6 | 45.22 | 90.93 | Mongolia |
| *Capsella orientalis* | OSBU2223 | CO2223 | A099co2223_5 | 45.22 | 90.93 | Mongolia |
| *Capsella orientalis* | OSBU2223 | CO2223 | A100co2223_10 | 45.22 | 90.93 | Mongolia |
| *Capsella orientalis* | OSBU2289 | CO2289 | A093co2289_1 | 51.78 | 81.18 | Altai |
| *Capsella orientalis* | OSBU2289 | CO2289 | A094co2289_2 | 51.78 | 81.18 | Altai |
| *Capsella orientalis* | OSBU2289 | CO2289 | A095co2289_7 | 51.78 | 81.18 | Altai |
| *Capsella orientalis* | OSBU2291 | CO2291 | A183co2291_7 | 51.13 | 81.09 | Altai |
| *Capsella orientalis* | OSBU2291 | CO2291 | A283co2291_2 | 53.32 | 57.58 | Altai |
| *Capsella orientalis* | OSBU2292 | CO2292 | A216co2292_1 | 50.40 | 80.22 | E_Kazakhstan |
| *Capsella orientalis* | OSBU2292 | CO2292 | A217co2292_8a | 50.40 | 80.22 | E_Kazakhstan |
| *Capsella orientalis* | OSBU2292 | CO2292 | A217co2292_8b | 50.40 | 80.22 | E_Kazakhstan |
| *Capsella orientalis* | OSBU2296 | CO2296 | A284co2296_9 | 45.66 | 80.27 | E_Kazakhstan |
| *Capsella orientalis* | OSBU2296 | CO2296 | A285co2296_8 | 45.66 | 80.27 | E_Kazakhstan |
| *Capsella orientalis* | OSBU2296 | CO2296 | A286co2296_5 | 45.66 | 80.27 | E_Kazakhstan |
| *Capsella orientalis* | OSBU2296 | CO2296 | A287co2296_7 | 45.66 | 80.27 | E_Kazakhstan |
| *Capsella orientalis* | OSBU2296 | CO2296 | A288co2296_6 | 45.66 | 80.27 | E_Kazakhstan |
| *Capsella orientalis* | OSBU2300 | CO2300 | A289co2300_2 | 49.85 | 82.42 | E_Kazakhstan |
| *Capsella orientalis* | OSBU2300 | CO2300 | A290co2300_1 | 49.85 | 82.42 | E_Kazakhstan |
| *Capsella orientalis* | OSBU2300 | CO2300 | A291co2300_4 | 49.85 | 82.42 | E_Kazakhstan |
| *Capsella orientalis* | OSBU2300 | CO2300 | A292co2300_3 | 49.85 | 82.42 | E_Kazakhstan |
| *Capsella orientalis* | OSBU2300 | CO2300 | A293co2300_5 | 49.85 | 82.42 | E_Kazakhstan |
| *Capsella orientalis* | OSBU2303 | CO2303 | A218co2303_4 | 50.09 | 82.40 | E_Kazakhstan |
| *Capsella orientalis* | OSBU2303 | CO2303 | A219co2303_5 | 50.09 | 82.40 | E_Kazakhstan |
| *Capsella orientalis* | OSBU2303 | CO2303 | A220co2303_6 | 50.09 | 82.40 | E_Kazakhstan |
| *Capsella orientalis* | OSBU2303 | CO2303 | A221co2303_1 | 50.09 | 82.40 | E_Kazakhstan |
| *Capsella orientalis* | OSBU2303 | CO2303 | A222co2303_3 | 50.09 | 82.40 | E_Kazakhstan |
| *Capsella orientalis* | OSBU2305 | CO2305 | A228co2305_5 | 48.77 | 82.36 | E_Kazakhstan |
| *Capsella orientalis* | OSBU2329 | CO2329 | A223co2329_6 | 49.24 | 73.03 | C_Kazakhstan |
| *Capsella orientalis* | OSBU2329 | CO2329 | A224co2329_1 | 49.24 | 73.03 | C_Kazakhstan |
| *Capsella orientalis* | OSBU2329 | CO2329 | A225co2329_2 | 49.24 | 73.03 | C_Kazakhstan |
| *Capsella orientalis* | OSBU2329 | CO2329 | A226co2329_5 | 49.24 | 73.03 | C_Kazakhstan |
| *Capsella orientalis* | OSBU2329 | CO2329 | A227co2329_3 | 49.24 | 73.03 | C_Kazakhstan |
| *Capsella orientalis* | OSBU2343 | CO2343 | A127co2343_1 | 51.09 | 66.47 | C_Kazakhstan |
| *Capsella orientalis* | OSBU2343 | CO2343 | A128co2343_3 | 51.09 | 66.47 | C_Kazakhstan |
| *Capsella orientalis* | OSBU2343 | CO2343 | A129co2343_2 | 51.09 | 66.47 | C_Kazakhstan |
| *Capsella orientalis* | OSBU2343 | CO2343 | A130co2343_5 | 51.09 | 66.47 | C_Kazakhstan |
| *Capsella orientalis* | OSBU2343 | CO2343 | A131co2343_4 | 51.09 | 66.47 | C_Kazakhstan |
| *Capsella orientalis* | OSBU2344 | CO2344 | A132co2344_8 | 51.82 | 68.36 | C_Kazakhstan |
| *Capsella orientalis* | OSBU2345 | CO2345 | A202co2345_2 | 51.53 | 69.81 | C_Kazakhstan |
| *Capsella orientalis* | OSBU2345 | CO2345 | A203co2345_5 | 51.53 | 69.81 | C_Kazakhstan |
| *Capsella orientalis* | OSBU2345 | CO2345 | A204co2345_9 | 51.53 | 69.81 | C_Kazakhstan |
| *Capsella orientalis* | OSBU2345 | CO2345 | A205co2345_7 | 51.53 | 69.81 | C_Kazakhstan |
| *Capsella orientalis* | OSBU2345 | CO2345 | A206co2345_2 | 51.53 | 69.81 | C_Kazakhstan |
| *Capsella orientalis* | OSBU2346 | CO2346 | A088co2346_5 | 52.99 | 78.65 | Altai |
| *Capsella orientalis* | OSBU2346 | CO2346 | A089co2346_2 | 52.99 | 78.65 | Altai |
| *Capsella orientalis* | OSBU2346 | CO2346 | A090co2346_3 | 52.99 | 78.65 | Altai |
| *Capsella orientalis* | OSBU2346 | CO2346 | A091co2346_4 | 52.99 | 78.65 | Altai |
| *Capsella orientalis* | OSBU2346 | CO2346 | A092co2346_1 | 52.99 | 78.65 | Altai |
| *Capsella orientalis* | OSBU2347 | CO2347 | A191co2347_3 | 52.49 | 78.13 | C_Kazakhstan |
| *Capsella orientalis* | OSBU2347 | CO2347 | A192co2347_2 | 52.49 | 78.13 | C_Kazakhstan |
| *Capsella orientalis* | OSBU2347 | CO2347 | A193co2347_5 | 52.49 | 78.13 | C_Kazakhstan |
| *Capsella orientalis* | OSBU2347 | CO2347 | A194co2347_4 | 52.49 | 78.13 | C_Kazakhstan |
| *Capsella orientalis* | OSBU2348 | CO2348 | A085co2348_1 | 51.89 | 77.06 | C_Kazakhstan |
| *Capsella orientalis* | OSBU2348 | CO2348 | A086co2348_6 | 51.89 | 77.06 | C_Kazakhstan |
| *Capsella orientalis* | OSBU2348 | CO2348 | A087co2348_5 | 51.89 | 77.06 | C_Kazakhstan |
| *Capsella orientalis* | OSBU2349 | CO2349 | A137co2349_6 | 51.76 | 72.41 | C_Kazakhstan |
| *Capsella orientalis* | OSBU2349 | CO2349 | A138co2349_7 | 51.76 | 72.41 | C_Kazakhstan |
| *Capsella orientalis* | OSBU2349 | CO2349 | A139co2349_5 | 51.76 | 72.41 | C_Kazakhstan |
| *Capsella orientalis* | OSBU2349 | CO2349 | A140co2349_1 | 51.76 | 72.41 | C_Kazakhstan |
| *Capsella orientalis* | OSBU2349 | CO2349 | A141co2349_3 | 51.76 | 72.41 | C_Kazakhstan |
| *Capsella orientalis* | OSBU2356 | CO2356 | A147co2356_1 | 50.40 | 80.22 | E_Kazakhstan |
| *Capsella orientalis* | OSBU2358 | CO2358 | A125co2358_2 | 47.40 | 80.61 | E_Kazakhstan |
| *Capsella orientalis* | OSBU2358 | CO2358 | A126co2358_1 | 47.40 | 80.61 | E_Kazakhstan |
| *Capsella orientalis* | OSBU2370 | CO2370 | A123co2370_3 | 51.83 | 55.71 | S_Ural |
| *Capsella orientalis* | OSBU2370 | CO2370 | A124co2370_1 | 51.83 | 55.71 | S_Ural |
| *Capsella orientalis* | OSBU2371 | CO2371 | A122co2371_1 | 51.85 | 56.34 | S_Ural |
| *Capsella orientalis* | OSBU2372 | CO2372 | A120co2372_5 | 51.79 | 56.36 | S_Ural |
| *Capsella orientalis* | OSBU2372 | CO2372 | A121co2372_2 | 51.79 | 56.36 | S_Ural |
| *Capsella orientalis* | OSBU2373 | CO2373 | A294co2373_5 | 51.48 | 57.37 | S_Ural |
| *Capsella orientalis* | OSBU2373 | CO2373 | A295co2373_7 | 51.48 | 57.37 | S_Ural |
| *Capsella orientalis* | OSBU2373 | CO2373 | A296co2373_10 | 51.48 | 57.37 | S_Ural |
| *Capsella orientalis* | OSBU2373 | CO2373 | A297co2373_8 | 51.48 | 57.37 | S_Ural |
| *Capsella orientalis* | OSBU2373 | CO2373 | A298co2373_9 | 51.48 | 57.37 | S_Ural |
| *Capsella orientalis* | OSBU2374 | CO2374 | A074co2374_1 | 51.51 | 57.59 | S_Ural |
| *Capsella orientalis* | OSBU2374 | CO2374 | A075co2374_3 | 51.51 | 57.59 | S_Ural |
| *Capsella orientalis* | OSBU2374 | CO2374 | A076co2374_2 | 51.51 | 57.59 | S_Ural |
| *Capsella orientalis* | OSBU2374 | CO2374 | A077co2374_4 | 51.51 | 57.59 | S_Ural |
| *Capsella orientalis* | OSBU2374 | CO2374 | A078co2374_6 | 51.51 | 57.59 | S_Ural |
| *Capsella orientalis* | OSBU2379 | CO2379 | A229co2379_4 | 51.49 | 53.37 | W_Kazakhstan |
| *Capsella orientalis* | OSBU2379 | CO2379 | A230co2379_5 | 51.49 | 53.37 | W_Kazakhstan |
| *Capsella orientalis* | OSBU2379 | CO2379 | A231co2379_6 | 51.49 | 53.37 | W_Kazakhstan |
| *Capsella orientalis* | OSBU2379 | CO2379 | A232co2379_1 | 51.49 | 53.37 | W_Kazakhstan |
| *Capsella orientalis* | OSBU2379 | CO2379 | A233co2379_2 | 51.49 | 53.37 | W_Kazakhstan |
| *Capsella orientalis* | OSBU2380 | CO2380 | A041co2380_2 | 51.37 | 53.15 | W_Kazakhstan |
| *Capsella orientalis* | OSBU2380 | CO2380 | A042co2380_4 | 51.37 | 53.15 | W_Kazakhstan |
| *Capsella orientalis* | OSBU2380 | CO2380 | A043co2380_1 | 51.37 | 53.15 | W_Kazakhstan |
| *Capsella orientalis* | OSBU2380 | CO2380 | A044co2380_5 | 51.37 | 53.15 | W_Kazakhstan |
| *Capsella orientalis* | OSBU2380 | CO2380 | A045co2380_3 | 51.37 | 53.15 | W_Kazakhstan |
| *Capsella orientalis* | OSBU2381 | CO2381 | A079co2381_6 | 50.86 | 53.17 | W_Kazakhstan |
| *Capsella orientalis* | OSBU2381 | CO2381 | A080co2381_4 | 50.86 | 53.17 | W_Kazakhstan |
| *Capsella orientalis* | OSBU2381 | CO2381 | A081co2381_5 | 50.86 | 53.17 | W_Kazakhstan |
| *Capsella orientalis* | OSBU2381 | CO2381 | A082co2381_2 | 50.86 | 53.17 | W_Kazakhstan |
| *Capsella orientalis* | OSBU2381 | CO2381 | A083co2381_1 | 50.86 | 53.17 | W_Kazakhstan |
| *Capsella orientalis* | OSBU2381 | CO2381 | A150co2381_8 | 50.86 | 53.17 | W_Kazakhstan |
| *Capsella orientalis* | OSBU2382 | CO2382 | A234co2382_5 | 50.26 | 52.61 | W_Kazakhstan |
| *Capsella orientalis* | OSBU2382 | CO2382 | A235co2382_3 | 50.26 | 52.61 | W_Kazakhstan |
| *Capsella orientalis* | OSBU2382 | CO2382 | A236co2382_6 | 50.26 | 52.61 | W_Kazakhstan |
| *Capsella orientalis* | OSBU2382 | CO2382 | A237co2382_1 | 50.26 | 52.61 | W_Kazakhstan |
| *Capsella orientalis* | OSBU2382 | CO2382 | A238co2382_2 | 50.26 | 52.61 | W_Kazakhstan |
| *Capsella orientalis* | OSBU2383 | CO2383 | A207co2383_3 | 50.24 | 57.25 | W_Kazakhstan |
| *Capsella orientalis* | OSBU2383 | CO2383 | A208co2383_1 | 50.24 | 57.25 | W_Kazakhstan |
| *Capsella orientalis* | OSBU2383 | CO2383 | A209co2383_4 | 50.24 | 57.25 | W_Kazakhstan |
| *Capsella orientalis* | OSBU2383 | CO2383 | A210co2383_6 | 50.24 | 57.25 | W_Kazakhstan |
| *Capsella orientalis* | OSBU2383 | CO2383 | A211co2383_2 | 50.24 | 57.25 | W_Kazakhstan |
| *Capsella orientalis* | OSBU2384 | CO2384 | A212co2384_5 | 48.83 | 58.13 | W_Kazakhstan |
| *Capsella orientalis* | OSBU2384 | CO2384 | A213co2384_3 | 48.83 | 58.13 | W_Kazakhstan |
| *Capsella orientalis* | OSBU2384 | CO2384 | A214co2384_2 | 48.83 | 58.13 | W_Kazakhstan |
| *Capsella orientalis* | OSBU2384 | CO2384 | A215co2384_4 | 48.83 | 58.13 | W_Kazakhstan |
| *Capsella orientalis* | OSBU2385 | CO2385 | A239co2385_3 | 48.76 | 58.54 | W_Kazakhstan |
| *Capsella orientalis* | OSBU2385 | CO2385 | A240co2385_5 | 48.76 | 58.54 | W_Kazakhstan |
| *Capsella orientalis* | OSBU2385 | CO2385 | A241co2385_4 | 48.76 | 58.54 | W_Kazakhstan |
| *Capsella orientalis* | OSBU2385 | CO2385 | A242co2385_6 | 48.76 | 58.54 | W_Kazakhstan |
| *Capsella orientalis* | OSBU2385 | CO2385 | A243co2385_1 | 48.76 | 58.54 | W_Kazakhstan |
| *Capsella orientalis* | OSBU2386 | CO2386 | A046co2386_2 | 48.93 | 59.11 | W_Kazakhstan |
| *Capsella orientalis* | OSBU2386 | CO2386 | A047co2386_3 | 48.93 | 59.11 | W_Kazakhstan |
| *Capsella orientalis* | OSBU2386 | CO2386 | A048co2386_4 | 48.93 | 59.11 | W_Kazakhstan |
| *Capsella orientalis* | OSBU2386 | CO2386 | A049co2386_5 | 48.93 | 59.11 | W_Kazakhstan |
| *Capsella orientalis* | OSBU2386 | CO2386 | A050co2386_1 | 48.93 | 59.11 | W_Kazakhstan |
| *Capsella orientalis* | OSBU2387 | CO2387 | A115co2387_4a | 49.97 | 60.06 | W_Kazakhstan |
| *Capsella orientalis* | OSBU2387 | CO2387 | A115co2387_4b | 49.97 | 60.06 | W_Kazakhstan |
| *Capsella orientalis* | OSBU2387 | CO2387 | A116co2387_3 | 49.97 | 60.06 | W_Kazakhstan |
| *Capsella orientalis* | OSBU2387 | CO2387 | A117co2387_6 | 49.97 | 60.06 | W_Kazakhstan |
| *Capsella orientalis* | OSBU2387 | CO2387 | A118co2387_2 | 49.97 | 60.06 | W_Kazakhstan |
| *Capsella orientalis* | OSBU2387 | CO2387 | A119co2387_5 | 49.97 | 60.06 | W_Kazakhstan |
| *Capsella orientalis* | OSBU2388 | CO2388 | A244co2388_1 | 50.98 | 61.66 | C_Kazakhstan |
| *Capsella orientalis* | OSBU2388 | CO2388 | A245co2388_2 | 50.98 | 61.66 | C_Kazakhstan |
| *Capsella orientalis* | OSBU2388 | CO2388 | A246co2388_5 | 50.98 | 61.66 | C_Kazakhstan |
| *Capsella orientalis* | OSBU2388 | CO2388 | A247co2388_6 | 50.98 | 61.66 | C_Kazakhstan |
| *Capsella orientalis* | OSBU2388 | CO2388 | A248co2388_3 | 50.98 | 61.66 | C_Kazakhstan |
| *Capsella orientalis* | OSBU2389 | CO2389 | A249co2389_9 | 51.69 | 61.57 | C_Kazakhstan |
| *Capsella orientalis* | OSBU2389 | CO2389 | A250co2389_4 | 51.69 | 61.57 | C_Kazakhstan |
| *Capsella orientalis* | OSBU2389 | CO2389 | A251co2389_3 | 51.69 | 61.57 | C_Kazakhstan |
| *Capsella orientalis* | OSBU2389 | CO2389 | A252co2389_2 | 51.69 | 61.57 | C_Kazakhstan |
| *Capsella orientalis* | OSBU2389 | CO2389 | A253co2389_5 | 51.69 | 61.57 | C_Kazakhstan |
| *Capsella orientalis* | OSBU2390 | CO2390 | A184co2390_2 | 53.23 | 63.60 | C_Kazakhstan |
| *Capsella orientalis* | OSBU2390 | CO2390 | A185co2390_5 | 53.23 | 63.60 | C_Kazakhstan |
| *Capsella orientalis* | OSBU2390 | CO2390 | A186co2390_4 | 53.23 | 63.60 | C_Kazakhstan |
| *Capsella orientalis* | OSBU2390 | CO2390 | A187co2390_3 | 53.23 | 63.60 | C_Kazakhstan |
| *Capsella orientalis* | OSBU2390 | CO2390 | A188co2390_7 | 53.23 | 63.60 | C_Kazakhstan |
| *Capsella orientalis* | OSBU2391 | CO2391 | A148co2391_12 | 53.30 | 57.52 | S_Ural |
| *Capsella orientalis* | OSBU2391 | CO2391 | A149co2391_13 | 53.30 | 57.52 | S_Ural |
| *Capsella orientalis* | OSBU2391 | CO2391 | A168co2391_8 | 53.30 | 57.52 | S_Ural |
| *Capsella orientalis* | OSBU2391 | CO2391 | A179co2391_7 | 53.32 | 57.59 | S_Ural |
| *Capsella orientalis* | OSBU2391 | CO2391 | A180co2391_2 | 53.32 | 57.59 | S_Ural |
| *Capsella orientalis* | OSBU2391 | CO2391 | A181co2391_5 | 53.32 | 57.59 | S_Ural |
| *Capsella orientalis* | OSBU2391 | CO2391 | A182co2391_4 | 53.32 | 57.59 | S_Ural |
| *Capsella orientalis* | OSBU2391 | CO2391 | A282co2391_1 | 53.30 | 57.52 | S_Ural |
| *Capsella orientalis* | OSBU2391 | CO2391 | A299co2391_14 | 53.30 | 57.52 | S_Ural |
| *Capsella orientalis* | OSBU2392 | CO2392 | A254co2392_2 | 53.00 | 67.17 | C_Kazakhstan |
| *Capsella orientalis* | OSBU2392 | CO2392 | A255co2392_5 | 53.00 | 67.17 | C_Kazakhstan |
| *Capsella orientalis* | OSBU2392 | CO2392 | A256co2392_1 | 53.00 | 67.17 | C_Kazakhstan |
| *Capsella orientalis* | OSBU2392 | CO2392 | A257co2392_3 | 53.00 | 67.17 | C_Kazakhstan |
| *Capsella orientalis* | OSBU2392 | CO2392 | A258co2392_4 | 53.00 | 67.17 | C_Kazakhstan |
| *Capsella orientalis* | OSBU2393 | CO2393 | A169co2393_3 | 53.27 | 68.07 | C_Kazakhstan |
| *Capsella orientalis* | OSBU2393 | CO2393 | A170co2393_1 | 53.27 | 68.07 | C_Kazakhstan |
| *Capsella orientalis* | OSBU2393 | CO2393 | A171co2393_5 | 53.27 | 68.07 | C_Kazakhstan |
| *Capsella orientalis* | OSBU2393 | CO2393 | A172co2393_4 | 53.27 | 68.07 | C_Kazakhstan |
| *Capsella orientalis* | OSBU2393 | CO2393 | A173co2393_2 | 53.27 | 68.07 | C_Kazakhstan |
| *Capsella orientalis* | OSBU2394 | CO2394 | A259co2394_1 | 52.69 | 70.29 | C_Kazakhstan |
| *Capsella orientalis* | OSBU2394 | CO2394 | A260co2394_6 | 52.69 | 70.29 | C_Kazakhstan |
| *Capsella orientalis* | OSBU2394 | CO2394 | A261co2394_5 | 52.69 | 70.29 | C_Kazakhstan |
| *Capsella orientalis* | OSBU2394 | CO2394 | A262co2394_4 | 52.69 | 70.29 | C_Kazakhstan |
| *Capsella orientalis* | OSBU2394 | CO2394 | A263co2394_2 | 52.69 | 70.29 | C_Kazakhstan |
| *Capsella orientalis* | OSBU2395 | CO2395 | A174co2395_4 | 51.69 | 74.57 | C_Kazakhstan |
| *Capsella orientalis* | OSBU2395 | CO2395 | A175co2395_2 | 51.69 | 74.57 | C_Kazakhstan |
| *Capsella orientalis* | OSBU2395 | CO2395 | A176co2395_5 | 51.69 | 74.57 | C_Kazakhstan |
| *Capsella orientalis* | OSBU2395 | CO2395 | A177co2395_3 | 51.69 | 74.57 | C_Kazakhstan |
| *Capsella orientalis* | OSBU2395 | CO2395 | A178co2395_1 | 51.69 | 74.57 | C_Kazakhstan |
| *Capsella orientalis* | OSBU2396 | CO2396 | A110co2396_5 | 51.82 | 79.66 | Altai |
| *Capsella orientalis* | OSBU2396 | CO2396 | A111co2396_4 | 51.82 | 79.66 | Altai |
| *Capsella orientalis* | OSBU2396 | CO2396 | A112co2396_2 | 51.82 | 79.66 | Altai |
| *Capsella orientalis* | OSBU2396 | CO2396 | A113co2396_1 | 51.82 | 79.66 | Altai |
| *Capsella orientalis* | OSBU2396 | CO2396 | A114co2396_3 | 51.82 | 79.66 | Altai |
| *Capsella orientalis* | OSBU2397 | CO2397 | A051co2397_2 | 52.02 | 80.37 | Altai |
| *Capsella orientalis* | OSBU2397 | CO2397 | A052co2397_1 | 52.02 | 80.37 | Altai |
| *Capsella orientalis* | OSBU2397 | CO2397 | A053co2397_5 | 52.02 | 80.37 | Altai |
| *Capsella orientalis* | OSBU2397 | CO2397 | A054co2397_4 | 52.02 | 80.37 | Altai |
| *Capsella orientalis* | OSBU2397 | CO2397 | A055co2397_6 | 52.02 | 80.37 | Altai |
| *Capsella orientalis* | OSBU2400 | CO2400 | A056co2400_2 | 52.50 | 82.73 | Altai |
| *Capsella orientalis* | OSBU2400 | CO2400 | A057co2400_5 | 52.50 | 82.73 | Altai |
| *Capsella orientalis* | OSBU2400 | CO2400 | A058co2400_4 | 52.50 | 82.73 | Altai |
| *Capsella orientalis* | OSBU2400 | CO2400 | A059co2400_8 | 52.50 | 82.73 | Altai |
| *Capsella orientalis* | OSBU2400 | CO2400 | A060co2400_3 | 52.50 | 82.73 | Altai |
| *Capsella orientalis* | OSBU26073 | CO26073 | A195co26073_3 | 48.08 | 106.84 | Mongolia |
| *Capsella orientalis* | OSBU26073 | CO26073 | A196co26073_1 | 48.08 | 106.84 | Mongolia |
| *Capsella orientalis* | OSBU26073 | CO26073 | A197co26073_4 | 48.08 | 106.84 | Mongolia |
| *Capsella orientalis* | OSBU26073 | CO26073 | A198co26073_2 | 48.08 | 106.84 | Mongolia |
| *Capsella orientalis* | OSBU26082 | CO26082 | A199co26082_2 | 48.12 | 106.90 | Mongolia |
| *Capsella orientalis* | OSBU26082 | CO26082 | A200co26082_1 | 48.12 | 106.90 | Mongolia |
| *Capsella orientalis* | OSBU26082 | CO26082 | A201co26082_3 | 48.12 | 106.90 | Mongolia |
| *Capsella orientalis* | OSBU26144 | CO26144 | A106co26144_4 | 48.31 | 98.92 | Mongolia |
| *Capsella orientalis* | OSBU26144 | CO26144 | A107co26144_5 | 48.31 | 98.92 | Mongolia |
| *Capsella orientalis* | OSBU26144 | CO26144 | A108co26144_3 | 48.31 | 98.92 | Mongolia |
| *Capsella orientalis* | OSBU26144 | CO26144 | A109co26144_6 | 48.31 | 98.92 | Mongolia |
| *Capsella rubella* | OSBU1335 | CR1335 | A037cr1335_1 | 41.08 | -8.06 | Mediterranean |
| *Capsella rubella* | OSBU2010 | CR2010 | A039cr2010_1 | 36.39 | 32.01 | Mediterranean |
| *Capsella rubella* | OSBU2207 | CR2207 | A038cr2207_2 | 43.77 | 11.23 | Mediterranean |
| *Capsella rubella* | OSBU2230 | CR2230 | A036cr2230_3 | 41.88 | 12.45 | Mediterranean |
| *Capsella rubella* | OSBU2239 | CR2239 | A035cr2239_3 | 36.26 | 27.95 | Mediterranean |
| *Capsella thracica* | OSBU2086 | CT2086 | A024ct2086_1 | 42.25 | 27.41 | Bulgaria |
| *Capsella thracica* | OSBU2086 | CT2086 | A025ct2086_2 | 42.25 | 27.41 | Bulgaria |
| *Capsella thracica* | OSBU2086 | CT2086 | A026ct2086_3 | 42.25 | 27.41 | Bulgaria |
| *Capsella thracica* | OSBU2090 | CT2090 | A027ct2090_10a | 42.05 | 27.56 | Bulgaria |
| *Capsella thracica* | OSBU2090 | CT2090 | A027ct2090_10b | 42.05 | 27.56 | Bulgaria |
| *Capsella thracica* | OSBU2090 | CT2090 | A028ct2090_8 | 42.05 | 27.56 | Bulgaria |
| *Capsella thracica* | OSBU2090 | CT2090 | A029ct2090_9 | 42.05 | 27.56 | Bulgaria |
| *Capsella thracica* | OSBU2101 | CT2101 | A030ct2101_1 | 42.12 | 24.40 | Bulgaria |
| *Capsella thracica* | OSBU2101 | CT2101 | A031ct2101_2 | 42.12 | 24.40 | Bulgaria |
| *Capsella thracica* | OSBU2101 | CT2101 | A032ct2101_3 | 42.12 | 24.40 | Bulgaria |
| *Capsella thracica* | OSBU2102 | CT2102 | A033ct2102_3 | 42.40 | 23.22 | Bulgaria |
| *Capsella thracica* | OSBU2102 | CT2102 | A034ct2102_2 | 42.40 | 23.22 | Bulgaria |
