# Supplementary material for: Pleistocene dynamics of the Eurasian steppe as a driving force of evolution: Phylogenetic history of the genus Capsella (Brassicaceae)
Source: Ecol Evol. 2021 Aug 18;11(18):12697–713. doi: 10.1002/ece3.8015 (PMC8462161; doi:10.1002/ece3.8015)
Supplement: Supplementary file 3 — File S3 [file ECE3-11-12697-s011.docx]

**Supplementary File 2: Statistics of the ipyrad output files** of different datasets under different parameters. ‘ori’ and ‘caps’ stand for ‘*Capsella orientalis*’ and ‘*Capsella’* datasets, respectively. ‘0.85’, ‘0.90’ and ‘0.95’ stand for the identity percentage above which the sequences are recognised as homologous. ’min50%’, ‘min25%’ and ‘min12.5%’ stand for the minimal percentage of samples with known data a given locus for it to be retained in the final alignment. ‘max20’, ‘max10’ and ‘max5’ stand for the maximum percentage of allowed SNPs per locus retained in the final alignment. The output files depicted in red represent the final datasets based on which all subsequent analyses were carried out.

| **ori_0_85_min50%_max20** | **total_filters** | **applied_order** | **retained_loci** |
| --- | --- | --- | --- |
| **total_prefiltered_loci** | 0 | 0 | 459062 |
| **filtered_by_rm_duplicates** | 4929 | 4929 | 454133 |
| **filtered_by_max_indels** | 45 | 45 | 454088 |
| **filtered_by_max_SNPs** | 6 | 5 | 454083 |
| **filtered_by_max_shared_het** | 359 | 357 | 453726 |
| **filtered_by_min_sample** | 448252 | 443809 | 9917 |
| **total_filtered_loci** | 453591 | 449145 | 9917 |
| **snps matrix size** | 235 | 10559 | 25.54% missing sites |
| **sequence matrix size** | 235 | 954590 | 9.61% missing sites |
|  |  |  |  |
| **ori_0_85_min25%_max20** | **total_filters** | **applied_order** | **retained_loci** |
| **total_prefiltered_loci** | 0 | 0 | 459062 |
| **filtered_by_rm_duplicates** | 4929 | 4929 | 454133 |
| **filtered_by_max_indels** | 68 | 68 | 454065 |
| **filtered_by_max_SNPs** | 126 | 122 | 453943 |
| **filtered_by_max_shared_het** | 2206 | 2196 | 451747 |
| **filtered_by_min_sample** | 438595 | 434505 | 17242 |
| **total_filtered_loci** | 445924 | 441820 | 17242 |
| **snps matrix size** | 235 | 32291 | 55.67% missing sites |
| **sequence matrix size** | 235 | 1597859 | 32.47% missing sites |
|  |  |  |  |
| **ori_0_85_min12.5%_max20** | **total_filters** | **applied_order** | **retained_loci** |
| **total_prefiltered_loci** | 0 | 0 | 459062 |
| **filtered_by_rm_duplicates** | 4929 | 4929 | 454133 |
| **filtered_by_max_indels** | 256 | 256 | 453877 |
| **filtered_by_max_SNPs** | 1160 | 1121 | 452756 |
| **filtered_by_max_shared_het** | 9146 | 9101 | 443655 |
| **filtered_by_min_sample** | 396554 | 393742 | 49913 |
| **total_filtered_loci** | 412045 | 409149 | 49913 |
| **snps matrix size** | 235 | 146361 | 79.30% missing sites |
| **sequence matrix size** | 235 | 4387096 | 66.41% missing sites |
|  |  |  |  |
| **ori_0_90_min50%_max20** | **total_filters** | **applied_order** | **retained_loci** |
| **total_prefiltered_loci** | 0 | 0 | 488997 |
| **filtered_by_rm_duplicates** | 6534 | 6534 | 482463 |
| **filtered_by_max_indels** | 21 | 21 | 482442 |
| **filtered_by_max_SNPs** | 8 | 7 | 482435 |
| **filtered_by_max_shared_het** | 390 | 387 | 482048 |
| **filtered_by_min_sample** | 477695 | 471961 | 10087 |
| **total_filtered_loci** | 484648 | 478910 | 10087 |
| **snps matrix size** | 235 | 9307 | 27.79% missing sites |
| **sequence matrix size** | 235 | 970069 | 9.28% missing sites |
|  |  |  |  |
| **ori_0_90_min25%_max20** | **total_filters** | **applied_order** | **retained_loci** |
| **total_prefiltered_loci** | 0 | 0 | 488997 |
| **filtered_by_rm_duplicates** | 6534 | 6534 | 482463 |
| **filtered_by_max_indels** | 30 | 30 | 482433 |
| **filtered_by_max_SNPs** | 31 | 30 | 482403 |
| **filtered_by_max_shared_het** | 2292 | 2285 | 480118 |
| **filtered_by_min_sample** | 467663 | 462414 | 17704 |
| **total_filtered_loci** | 476550 | 471293 | 17704 |
| **snps matrix size** | 235 | 31126 | 57.34% missing sites |
| **sequence matrix size** | 235 | 1638302 | 32.52% missing sites |
|  |  |  |  |
| **ori_0_90_min12.5%_max20** | **total_filters** | **applied_order** | **retained_loci** |
| **total_prefiltered_loci** | 0 | 0 | 488997 |
| **filtered_by_rm_duplicates** | 6534 | 6534 | 482463 |
| **filtered_by_max_indels** | 106 | 106 | 482357 |
| **filtered_by_max_SNPs** | 280 | 273 | 482084 |
| **filtered_by_max_shared_het** | 9596 | 9570 | 472514 |
| **filtered_by_min_sample** | 424253 | 420634 | 51880 |
| **total_filtered_loci** | 440769 | 437117 | 51880 |
| **snps matrix size** | 235 | 137104 | 79.52% missing sites |
| **sequence matrix size** | 235 | 4563271 | 66.72% missing sites |
|  |  |  |  |
| **ori_0_95_min50%_max20** | **total_filters** | **applied_order** | **retained_loci** |
| **total_prefiltered_loci** | 0 | 0 | 530773 |
| **filtered_by_rm_duplicates** | 9970 | 9970 | 520803 |
| **filtered_by_max_indels** | 9 | 9 | 520794 |
| **filtered_by_max_SNPs** | 3 | 1 | 520793 |
| **filtered_by_max_shared_het** | 336 | 336 | 520457 |
| **filtered_by_min_sample** | 519056 | 510374 | 10083 |
| **total_filtered_loci** | 529374 | 520690 | 10083 |
| **snps matrix size** | 235 | 5527 | 27.55% missing sites |
| **sequence matrix size** | 235 | 966425 | 9.14% missing sites |
|  |  |  |  |
| **ori_0_95_min50%_max10** | **total_filters** | **applied_order** | **retained_loci** |
| **total_prefiltered_loci** | 0 | 0 | 639291 |
| **filtered_by_rm_duplicates** | 9055 | 9055 | 630236 |
| **filtered_by_max_indels** | 8 | 8 | 630228 |
| **filtered_by_max_SNPs** | 45 | 43 | 630185 |
| **filtered_by_max_shared_het** | 349 | 329 | 629856 |
| **filtered_by_min_sample** | 619734 | 619734 | 10122 |
| **total_filtered_loci** | 629191 | 629169 | 10122 |
| **snps matrix size** | 235 | 5361 | 26.73% missing sites |
| **sequence matrix size** | 235 | 970507 | 9.10% missing sites |
|  |  |  |  |
| **ori_0_95_min50%_max5** | **total_filters** | **applied_order** | **retained_loci** |
| **total_prefiltered_loci** | 0 | 0 | 639291 |
| **filtered_by_rm_duplicates** | 9055 | 9055 | 630236 |
| **filtered_by_max_indels** | 8 | 8 | 630228 |
| **filtered_by_max_SNPs** | 232 | 228 | 630000 |
| **filtered_by_max_shared_het** | 349 | 262 | 629738 |
| **filtered_by_min_sample** | 619734 | 619734 | 10004 |
| **total_filtered_loci** | 629378 | 629287 | 10004 |
| **snps matrix size** | 235 | 4644 | 24.23% missing sites |
| **sequence matrix size** | 235 | 959279 | 8.79% missing sites |
|  |  |  |  |
| **ori_0_95_min25%_max20** | **total_filters** | **applied_order** | **retained_loci** |
| **total_prefiltered_loci** | 0 | 0 | 530773 |
| **filtered_by_rm_duplicates** | 9970 | 9970 | 520803 |
| **filtered_by_max_indels** | 17 | 17 | 520786 |
| **filtered_by_max_SNPs** | 6 | 3 | 520783 |
| **filtered_by_max_shared_het** | 2119 | 2118 | 518665 |
| **filtered_by_min_sample** | 508720 | 500934 | 17731 |
| **total_filtered_loci** | 520832 | 513042 | 17731 |
| **snps matrix size** | 235 | 19399 | 57.84% missing sites |
| **sequence matrix size** | 235 | 1634399 | 32.31% missing sites |
|  |  |  |  |
| **ori_0_95_min12.5%_max20** | **total_filters** | **applied_order** | **retained_loci** |
| **total_prefiltered_loci** | 0 | 0 | 530773 |
| **filtered_by_rm_duplicates** | 9970 | 9970 | 520803 |
| **filtered_by_max_indels** | 42 | 42 | 520761 |
| **filtered_by_max_SNPs** | 23 | 19 | 520742 |
| **filtered_by_max_shared_het** | 8802 | 8796 | 511946 |
| **filtered_by_min_sample** | 465607 | 460544 | 51402 |
| **total_filtered_loci** | 484444 | 479371 | 51402 |
| **snps matrix size** | 235 | 85391 | 79.99% missing sites |
| **sequence matrix size** | 235 | 4506529 | 66.52% missing sites |
|  |  |  |  |
|  |  |  |  |
|  |  |  |  |
| **caps_0_85_min50%_max20** | **total_filters** | **applied_order** | **retained_loci** |
| **total_prefiltered_loci** | 0 | 0 | 589890 |
| **filtered_by_rm_duplicates** | 4899 | 4899 | 584991 |
| **filtered_by_max_indels** | 327 | 327 | 584664 |
| **filtered_by_max_SNPs** | 46 | 40 | 584624 |
| **filtered_by_max_shared_het** | 307 | 298 | 584326 |
| **filtered_by_min_sample** | 575275 | 575275 | 9051 |
| **total_filtered_loci** | 580854 | 580839 | 9051 |
| **snps matrix size** | 282 | 48183 | 14.37% missing sites |
| **sequence matrix size** | 282 | 874991 | 12.64% missing sites |
|  |  |  |  |
| **caps_0_85_min25%_max20** | **total_filters** | **applied_order** | **retained_loci** |
| **total_prefiltered_loci** | 0 | 0 | 589890 |
| **filtered_by_rm_duplicates** | 4899 | 4899 | 584991 |
| **filtered_by_max_indels** | 394 | 394 | 584597 |
| **filtered_by_max_SNPs** | 123 | 114 | 584483 |
| **filtered_by_max_shared_het** | 2139 | 2116 | 582367 |
| **filtered_by_min_sample** | 566344 | 566344 | 16023 |
| **total_filtered_loci** | 573899 | 573867 | 16023 |
| **snps matrix size** | 282 | 74024 | 33.52% missing sites |
| **sequence matrix size** | 282 | 1491829 | 34.91% missing sites |
|  |  |  |  |
| **caps_0_85_min12.5%_max20** | **total_filters** | **applied_order** | **retained_loci** |
| **total_prefiltered_loci** | 0 | 0 | 589890 |
| **filtered_by_rm_duplicates** | 4899 | 4899 | 584991 |
| **filtered_by_max_indels** | 586 | 586 | 584405 |
| **filtered_by_max_SNPs** | 597 | 577 | 583828 |
| **filtered_by_max_shared_het** | 7472 | 7425 | 576403 |
| **filtered_by_min_sample** | 537635 | 537635 | 38768 |
| **total_filtered_loci** | 551189 | 551122 | 38768 |
| **snps matrix size** | 282 | 155274 | 60.46% missing sites |
| **sequence matrix size** | 282 | 3472199 | 63.14% missing sites |
|  |  |  |  |
| **caps_0_90_min50%_max20** | **total_filters** | **applied_order** | **retained_loci** |
| **total_prefiltered_loci** | 0 | 0 | 630504 |
| **filtered_by_rm_duplicates** | 6797 | 6797 | 623707 |
| **filtered_by_max_indels** | 112 | 112 | 623595 |
| **filtered_by_max_SNPs** | 19 | 17 | 623578 |
| **filtered_by_max_shared_het** | 312 | 305 | 623273 |
| **filtered_by_min_sample** | 614146 | 614146 | 9127 |
| **total_filtered_loci** | 621386 | 621377 | 9127 |
| **snps matrix size** | 282 | 43222 | 13.74% missing sites |
| **sequence matrix size** | 282 | 881075 | 12.28% missing sites |
|  |  |  |  |
| **caps_0_90_min25%_max20** | **total_filters** | **applied_order** | **retained_loci** |
| **total_prefiltered_loci** | 0 | 0 | 630504 |
| **filtered_by_rm_duplicates** | 6797 | 6797 | 623707 |
| **filtered_by_max_indels** | 140 | 140 | 623567 |
| **filtered_by_max_SNPs** | 44 | 41 | 623526 |
| **filtered_by_max_shared_het** | 2201 | 2188 | 621338 |
| **filtered_by_min_sample** | 605005 | 605005 | 16333 |
| **total_filtered_loci** | 614187 | 614171 | 16333 |
| **snps matrix size** | 282 | 68055 | 34.02% missing sites |
| **sequence matrix size** | 282 | 1518922 | 35.00% missing sites |
|  |  |  |  |
| **caps_0_90_min12.5%_max20** | **total_filters** | **applied_order** | **retained_loci** |
| **total_prefiltered_loci** | 0 | 0 | 630504 |
| **filtered_by_rm_duplicates** | 6797 | 6797 | 623707 |
| **filtered_by_max_indels** | 219 | 219 | 623488 |
| **filtered_by_max_SNPs** | 137 | 132 | 623356 |
| **filtered_by_max_shared_het** | 7771 | 7744 | 615612 |
| **filtered_by_min_sample** | 575561 | 575561 | 40051 |
| **total_filtered_loci** | 590485 | 590453 | 40051 |
| **snps matrix size** | 282 | 144525 | 61.00% missing sites |
| **sequence matrix size** | 282 | 3590201 | 63.49% missing sites |
|  |  |  |  |
| **caps_0_95_min50%_max20** | **total_filters** | **applied_order** | **retained_loci** |
| **total_prefiltered_loci** | 0 | 0 | 692730 |
| **filtered_by_rm_duplicates** | 12162 | 12162 | 680568 |
| **filtered_by_max_indels** | 17 | 17 | 680551 |
| **filtered_by_max_SNPs** | 4 | 4 | 680547 |
| **filtered_by_max_shared_het** | 290 | 289 | 680258 |
| **filtered_by_min_sample** | 671956 | 671956 | 8302 |
| **total_filtered_loci** | 684429 | 684428 | 8302 |
| **snps matrix size** | 282 | 26589 | 14.12% missing sites |
| **sequence matrix size** | 282 | 800204 | 13.06% missing sites |
|  |  |  |  |
| **caps_0_95_min50%_max10** | **total_filters** | **applied_order** | **retained_loci** |
| **total_prefiltered_loci** | 0 | 0 | 692730 |
| **filtered_by_rm_duplicates** | 12162 | 12162 | 680568 |
| **filtered_by_max_indels** | 17 | 17 | 680551 |
| **filtered_by_max_SNPs** | 207 | 206 | 680345 |
| **filtered_by_max_shared_het** | 290 | 259 | 680086 |
| **filtered_by_min_sample** | 671956 | 671956 | 8130 |
| **total_filtered_loci** | 684632 | 684600 | 8130 |
| **snps matrix size** | 282 | 24584 | 13.47% missing sites |
| **sequence matrix size** | 282 | 783041 | 12.92% missing sites |
|  |  |  |  |
| **caps_0_95_min50%_max5** | **total_filters** | **applied_order** | **retained_loci** |
| **total_prefiltered_loci** | 0 | 0 | 692730 |
| **filtered_by_rm_duplicates** | 12162 | 12162 | 680568 |
| **filtered_by_max_indels** | 17 | 17 | 680551 |
| **filtered_by_max_SNPs** | 2063 | 2058 | 678493 |
| **filtered_by_max_shared_het** | 290 | 188 | 678305 |
| **filtered_by_min_sample** | 671956 | 671956 | 6349 |
| **total_filtered_loci** | 686488 | 686381 | 6349 |
| **snps matrix size** | 282 | 12635 | 13.17% missing sites |
| **sequence matrix size** | 282 | 605569 | 13.01% missing sites |
|  |  |  |  |
| **caps_0_95_min25%_max20** | **total_filters** | **applied_order** | **retained_loci** |
| **total_prefiltered_loci** | 0 | 0 | 692730 |
| **filtered_by_rm_duplicates** | 12162 | 12162 | 680568 |
| **filtered_by_max_indels** | 24 | 24 | 680544 |
| **filtered_by_max_SNPs** | 12 | 11 | 680533 |
| **filtered_by_max_shared_het** | 2162 | 2159 | 678374 |
| **filtered_by_min_sample** | 662725 | 662725 | 15649 |
| **total_filtered_loci** | 677085 | 677081 | 15649 |
| **snps matrix size** | 282 | 44253 | 35.81% missing sites |
| **sequence matrix size** | 282 | 1445668 | 36.65% missing sites |
|  |  |  |  |
| **caps_0_95_min12.5%_max20** | **total_filters** | **applied_order** | **retained_loci** |
| **total_prefiltered_loci** | 0 | 0 | 692730 |
| **filtered_by_rm_duplicates** | 12162 | 12162 | 680568 |
| **filtered_by_max_indels** | 43 | 43 | 680525 |
| **filtered_by_max_SNPs** | 19 | 16 | 680509 |
| **filtered_by_max_shared_het** | 7524 | 7519 | 672990 |
| **filtered_by_min_sample** | 634033 | 634033 | 38957 |
| **total_filtered_loci** | 653781 | 653773 | 38957 |
| **snps matrix size** | 282 | 93512 | 61.76% missing sites |
| **sequence matrix size** | 282 | 3474959 | 64.52% missing sites |
