# Supplementary material for: Pleistocene dynamics of the Eurasian steppe as a driving force of evolution: Phylogenetic history of the genus Capsella (Brassicaceae)
Source: Ecol Evol. 2021 Aug 18;11(18):12697–713. doi: 10.1002/ece3.8015 (PMC8462161; doi:10.1002/ece3.8015)
Supplement: Supplementary file 12 — File S12 [file ECE3-11-12697-s001.docx]

**Supplementary File 12-1: Molecular diversity indexes analysis** corrected per populations size.

| **Statistics** |  |  |  |  | **Co_2380_N_KZH** | **Co_2386_S_KZH** | **Co2397_Altai** | **Co_2400_Altai** | **Co_1980_Altai** | **Co_1979_Altai** |
| --- | --- | --- | --- | --- | --- | --- | --- | --- | --- | --- |
|  |  |  |  |  |  |  |  |  |  |  |
| **No.** | **of** | **gene** | **copies** |  | 10 | 10 | 10 | 10 | 10 | 6 |
| **No.** | **of** | **loci** |  |  | 4644 | 4644 | 4644 | 4644 | 4644 | 4644 |
| **No.** | **of** | **usable** | **loci** |  | 2319 | 2302 | 2570 | 2809 | 2585 | 3810 |
| **No.** | **of** | **polym.** | **loci** |  | 217 | 378 | 257 | 637 | 38 | 387 |
|  |  |  |  |  |  |  |  |  |  |  |
|  |  |  |  |  |  |  |  |  |  |  |
| **Molecular div ind** |  |  |  |  |  |  |  |  |  |  |
|  |  |  |  |  |  |  |  |  |  |  |
| **Statistics** |  |  |  |  | **Co_2380_N_KZH** | **Co_2386_S_KZH** | **Co2397_Altai** | **Co_2400_Altai** | **Co_1980_Altai** | **Co_1979_Altai** |
|  |  |  |  |  |  |  |  |  |  |  |
|  | **No.** | **of** | **transitions** |  | 126 | 220 | 146 | 353 | 17 | 232 |
|  | **No.** | **of** | **transversions** |  | 91 | 159 | 111 | 285 | 22 | 156 |
|  | **No.** | **of** | **substitutions** |  | 217 | 379 | 257 | 638 | 39 | 388 |
|  | **No.** | **of** | **indels** |  | 97 | 84 | 76 | 100 | 90 | 167 |
|  | **No.** | **of** | **ts.** | **sites** | 126 | 220 | 146 | 353 | 17 | 232 |
|  | **No.** | **of** | **tv.** | **sites** | 91 | 159 | 111 | 285 | 22 | 156 |
|  | **No.** | **of** | **subst.** | **sites** | 217 | 378 | 257 | 637 | 38 | 387 |
|  | **No.** | **private** | **subst.** | **sites** | 27 | 55 | 10 | 20 | 6 | 13 |
|  | **No.** | **of** | **indel** | **sites** | 97 | 84 | 76 | 100 | 90 | 167 |
|  | **Pi** |  |  |  | 111,689 | 187,356 | 138,156 | 238,689 | 48,8 | 270,533 |
|  |  |  |  |  |  |  |  |  |  |  |
|  | **Theta_S** |  |  |  | 76,70641 | 133,61762 | 90,84584 | 225,17043 | 13,43246 | 169,48905 |
|  | **s.d.** | **Theta_S** |  |  | 31,17888 | 54,04036 | 36,85886 | 90,81655 | 5,75284 | 79,71612 |
|  | **Theta_pi** |  |  |  | 111,68889 | 187,35556 | 138,15556 | 238,68889 | 48,8 | 270,53333 |
|  | **s.d.** | **Theta_pi** |  |  | 59,38027 | 99,34913 | 73,36063 | 126,46439 | 26,15975 | 156,59612 |
|  |  |  |  |  |  |  |  |  |  |  |
|  | **No** | **subs** | **sites** | **gene copy** | 21,70 | 80 | 25,70 | 63,70 | 3,80 | 64,50 |
|  | **No** | **priv subs** | **sites** | **gene copy** | 2,70 | 0 | 1,00 | 2,00 | 0,60 | 2,17 |

| **Co_1933_Mongolia** | **Co2374_N_KZH** | **Co_2381_S_KZH** | **Co_1978_Altai** | **Co_2348_S_KZH** | **Co_2346_N_KZH** | **Co_2289_Altai** | **Co_2223_Mongolia** | **Co_1985_Altai** |
| --- | --- | --- | --- | --- | --- | --- | --- | --- |
|  |  |  |  |  |  |  |  |  |
| 10 | 10 | 12 | 2 | 6 | 10 | 6 | 10 | 10 |
| 4644 | 4644 | 4644 | 4644 | 4644 | 4644 | 4644 | 4644 | 4644 |
| 2343 | 2376 | 2452 | 4283 | 3317 | 2277 | 2289 | 2021 | 2197 |
| 106 | 349 | 408 | 81 | 184 | 442 | 310 | 68 | 134 |
|  |  |  |  |  |  |  |  |  |
|  |  |  |  |  |  |  |  |  |
|  |  |  |  |  |  |  |  |  |
|  |  |  |  |  |  |  |  |  |
| **Co_1933_Mongolia** | **Co2374_N_KZH** | **Co_2381_S_KZH** | **Co_1978_Altai** | **Co_2348_S_KZH** | **Co_2346_N_KZH** | **Co_2289_Altai** | **Co_2223_Mongolia** | **Co_1985_Altai** |
|  |  |  |  |  |  |  |  |  |
| 49 | 196 | 240 | 42 | 103 | 252 | 154 | 36 | 74 |
| 57 | 153 | 168 | 39 | 81 | 191 | 156 | 32 | 60 |
| 106 | 349 | 408 | 81 | 184 | 443 | 310 | 68 | 134 |
| 91 | 157 | 88 | 0 | 77 | 132 | 153 | 64 | 104 |
| 49 | 196 | 240 | 42 | 103 | 252 | 154 | 36 | 74 |
| 57 | 153 | 168 | 39 | 81 | 191 | 156 | 32 | 60 |
| 106 | 349 | 408 | 81 | 184 | 442 | 310 | 68 | 134 |
| 23 | 25 | 55 | 6 | 7 | 26 | 46 | 12 | 11 |
| 91 | 157 | 88 | 0 | 77 | 132 | 153 | 64 | 104 |
| 67,8 | 195,467 | 185,303 | 81 | 135,2 | 220 | 225,2 | 46,556 | 94,222 |
|  |  |  |  |  |  |  |  |  |
| 37,46949 | 123,36653 | 135,10482 | 81 | 80,58394 | 156,24071 | 135,76642 | 24,03703 | 47,36709 |
| 15,41564 | 49,92252 | 51,97728 | 57,62812 | 38,09142 | 63,12797 | 63,92758 | 10,0176 | 19,39229 |
| 67,8 | 195,46667 | 185,30303 | 81 | 135,2 | 220 | 225,2 | 46,55556 | 94,22222 |
| 36,19665 | 103,63359 | 96,25109 | 81,49847 | 78,46086 | 116,59257 | 130,4228 | 24,97407 | 50,15387 |
|  |  |  |  |  |  |  |  |  |
| 10,60 | 34,90 | 34,00 | 40,50 | 30,67 | 44,20 | 51,67 | 6,80 | 13,40 |
| 2,30 | 2,50 | 4,58 | 3,00 | 1,17 | 2,60 | 7,67 | 1,20 | 1,10 |

| **Co_26144_Mongolia** | **Co_2396_Altai** | **Co_2387_S_KZH** | **Co_2372_N_KZH** | **Co_2371_N_KZH** | **Co_2370_N_KZH** | **Co_2358_E_KZH** | **Co_2343_S_KZH** | **Co_2344_S_KZH** |
| --- | --- | --- | --- | --- | --- | --- | --- | --- |
|  |  |  |  |  |  |  |  |  |
| 8 | 10 | 12 | 4 | 2 | 4 | 4 | 10 | 2 |
| 4644 | 4644 | 4644 | 4644 | 4644 | 4644 | 4644 | 4644 | 4644 |
| 2785 | 2958 | 2410 | 2149 | 3619 | 3301 | 2793 | 3169 | 3370 |
| 204 | 320 | 199 | 161 | 57 | 238 | 145 | 437 | 60 |
|  |  |  |  |  |  |  |  |  |
|  |  |  |  |  |  |  |  |  |
|  |  |  |  |  |  |  |  |  |
|  |  |  |  |  |  |  |  |  |
| **Co_26144_Mongolia** | **Co_2396_Altai** | **Co_2387_S_KZH** | **Co_2372_N_KZH** | **Co_2371_N_KZH** | **Co_2370_N_KZH** | **Co_2358_E_KZH** | **Co_2343_S_KZH** | **Co_2344_S_KZH** |
|  |  |  |  |  |  |  |  |  |
| 108 | 196 | 110 | 99 | 32 | 128 | 82 | 243 | 28 |
| 97 | 124 | 89 | 62 | 25 | 111 | 63 | 195 | 32 |
| 205 | 320 | 199 | 161 | 57 | 239 | 145 | 438 | 60 |
| 144 | 164 | 119 | 91 | 0 | 72 | 79 | 107 | 0 |
| 108 | 196 | 110 | 99 | 32 | 128 | 82 | 242 | 28 |
| 97 | 124 | 89 | 62 | 25 | 111 | 63 | 195 | 32 |
| 204 | 320 | 199 | 161 | 57 | 238 | 145 | 437 | 60 |
| 17 | 18 | 30 | 12 | 0 | 14 | 12 | 53 | 7 |
| 144 | 164 | 119 | 91 | 0 | 72 | 79 | 107 | 0 |
| 130,393 | 171,667 | 117,424 | 164,833 | 57 | 203 | 145,167 | 214,978 | 60 |
|  |  |  |  |  |  |  |  |  |
| 78,67769 | 113,11544 | 65,89672 | 87,81818 | 57 | 129,81818 | 79,09091 | 154,47328 | 60 |
| 34,07666 | 45,80465 | 25,53926 | 47,50762 | 40,6571 | 70,05684 | 42,82201 | 62,418 | 42,7785 |
| 130,39286 | 171,66667 | 117,42424 | 164,83333 | 57 | 203 | 145,16667 | 214,97778 | 60 |
| 71,52685 | 91,06194 | 61,13188 | 107,99997 | 57,49783 | 132,90884 | 95,1648 | 113,93974 | 60,49793 |
|  |  |  |  |  |  |  |  |  |
| 25,50 | 32,00 | 16,58 | 40,25 | 28,50 | 59,50 | 36,25 | 43,70 | 30,00 |
| 2,13 | 1,80 | 2,50 | 3,00 | 0,00 | 3,50 | 3,00 | 5,30 | 3,50 |

| **Co_2349_N_KZH** | **Co_2356_Altai** | **Co_2393_N_KZH** | **Co_2395_S_KZH** | **Co_2391_N_KZH** | **Co_2291_Altai** | **Co_2390_N_KZH** | **Co_1939_N_KZH** | **Co_1940_Altai** |
| --- | --- | --- | --- | --- | --- | --- | --- | --- |
|  |  |  |  |  |  |  |  |  |
| 10 | 2 | 10 | 10 | 10 | 4 | 10 | 2 | 2 |
| 4644 | 4644 | 4644 | 4644 | 4644 | 4644 | 4644 | 4644 | 4644 |
| 774 | 3582 | 1540 | 2851 | 2328 | 3615 | 2133 | 3427 | 3525 |
| 5 | 20 | 270 | 559 | 155 | 318 | 395 | 25 | 32 |
|  |  |  |  |  |  |  |  |  |
|  |  |  |  |  |  |  |  |  |
|  |  |  |  |  |  |  |  |  |
|  |  |  |  |  |  |  |  |  |
| **Co_2349_N_KZH** | **Co_2356_Altai** | **Co_2393_N_KZH** | **Co_2395_S_KZH** | **Co_2391_N_KZH** | **Co_2291_Altai** | **Co_2390_N_KZH** | **Co_1939_N_KZH** | **Co_1940_Altai** |
|  |  |  |  |  |  |  |  |  |
| 5 | 10 | 161 | 255 | 81 | 169 | 224 | 12 | 15 |
| 0 | 10 | 110 | 305 | 75 | 149 | 171 | 13 | 17 |
| 5 | 20 | 271 | 560 | 156 | 318 | 395 | 25 | 32 |
| 14 | 0 | 43 | 189 | 98 | 133 | 67 | 0 | 0 |
| 5 | 10 | 160 | 254 | 81 | 169 | 224 | 12 | 15 |
| 0 | 10 | 110 | 305 | 75 | 149 | 171 | 13 | 17 |
| 5 | 20 | 270 | 559 | 155 | 318 | 395 | 25 | 32 |
| 0 | 1 | 28 | 186 | 12 | 36 | 28 | 1 | 1 |
| 14 | 0 | 43 | 189 | 98 | 133 | 67 | 0 | 0 |
| 7,356 | 20 | 127,178 | 272,2 | 101,422 | 265,5 | 171,978 | 25 | 32 |
|  |  |  |  |  |  |  |  |  |
| 1,76743 | 20 | 95,44116 | 197,59854 | 54,79029 | 173,45455 | 139,62688 | 25 | 32 |
| 1,0139 | 14,49138 | 38,70483 | 79,74115 | 22,37457 | 93,48436 | 56,45426 | 18,02776 | 22,97825 |
| 7,35556 | 20 | 127,17778 | 272,2 | 101,42222 | 265,5 | 171,97778 | 25 | 32 |
| 4,25352 | 20,4939 | 67,56191 | 144,16558 | 53,95713 | 173,69837 | 91,22628 | 25,4951 | 32,49615 |
|  |  |  |  |  |  |  |  |  |
| 0,50 | 10,00 | 27,00 | 55,90 | 15,50 | 79,50 | 39,50 | 12,50 | 16,00 |
| 0,00 | 0,50 | 2,80 | 18,60 | 1,20 | 9,00 | 2,80 | 0,50 | 0,50 |

| **Co_2347_N_KZH** | **Co_26073_Mongolia** | **Co_26082_Mongolia** | **Co_2345_S_KZH** | **Co_2383_S_KZH** | **Co_2384_S_KZH** | **Co_2292_Altai** | **Co_2303_E_KZH** | **Co_2329_S_KZH** |
| --- | --- | --- | --- | --- | --- | --- | --- | --- |
|  |  |  |  |  |  |  |  |  |
| 8 | 8 | 6 | 10 | 10 | 8 | 6 | 10 | 10 |
| 4644 | 4644 | 4644 | 4644 | 4644 | 4644 | 4644 | 4644 | 4644 |
| 2666 | 3013 | 2898 | 2535 | 1951 | 2416 | 2197 | 2405 | 1622 |
| 165 | 34 | 29 | 388 | 143 | 327 | 254 | 209 | 204 |
|  |  |  |  |  |  |  |  |  |
|  |  |  |  |  |  |  |  |  |
|  |  |  |  |  |  |  |  |  |
|  |  |  |  |  |  |  |  |  |
| **Co_2347_N_KZH** | **Co_26073_Mongolia** | **Co_26082_Mongolia** | **Co_2345_S_KZH** | **Co_2383_S_KZH** | **Co_2384_S_KZH** | **Co_2292_Altai** | **Co_2303_E_KZH** | **Co_2329_S_KZH** |
|  |  |  |  |  |  |  |  |  |
| 88 | 12 | 9 | 212 | 80 | 171 | 154 | 108 | 115 |
| 77 | 22 | 20 | 177 | 63 | 156 | 100 | 102 | 89 |
| 165 | 34 | 29 | 389 | 143 | 327 | 254 | 210 | 204 |
| 101 | 85 | 94 | 108 | 50 | 117 | 117 | 104 | 46 |
| 88 | 12 | 9 | 212 | 80 | 171 | 154 | 108 | 115 |
| 77 | 22 | 20 | 177 | 63 | 156 | 100 | 102 | 89 |
| 165 | 34 | 29 | 388 | 143 | 327 | 254 | 209 | 204 |
| 11 | 3 | 4 | 38 | 18 | 57 | 29 | 13 | 37 |
| 101 | 85 | 94 | 108 | 50 | 117 | 117 | 104 | 46 |
| 112,929 | 52,464 | 63,6 | 193,244 | 68,978 | 195,536 | 185,667 | 113,289 | 81,756 |
|  |  |  |  |  |  |  |  |  |
| 63,63636 | 13,11295 | 12,70073 | 137,15248 | 50,54846 | 126,1157 | 111,24088 | 73,87852 | 72,1111 |
| 27,63141 | 5,97468 | 6,30088 | 55,4603 | 20,67043 | 54,40327 | 52,44493 | 30,04287 | 29,33286 |
| 112,92857 | 52,46429 | 63,6 | 193,24444 | 68,97778 | 195,53571 | 185,66667 | 113,28889 | 81,75556 |
| 61,99913 | 29,01175 | 37,12142 | 102,45976 | 36,8188 | 107,06555 | 107,59809 | 60,22544 | 43,56855 |
|  |  |  |  |  |  |  |  |  |
| 20,63 | 4,25 | 4,83 | 38,80 | 14,30 | 40,88 | 42,33 | 20,90 | 20,40 |
| 1,38 | 0,38 | 0,67 | 3,80 | 1,80 | 7,13 | 4,83 | 1,30 | 3,70 |

| **Co_2305_E_KZH** | **Co_2379_N_KZH** | **Co_2382_S_KZH** | **Co_2385_S_KZH** | **Co_2388_S_KZH** | **Co_2389_N_KZH** | **Co_2392_N_KZH** | **Co_2394_N_KZH** | **Co_1981_Altai** |
| --- | --- | --- | --- | --- | --- | --- | --- | --- |
|  |  |  |  |  |  |  |  |  |
| 2 | 10 | 10 | 10 | 10 | 10 | 10 | 10 | 10 |
| 4644 | 4644 | 4644 | 4644 | 4644 | 4644 | 4644 | 4644 | 4644 |
| 3238 | 2342 | 1883 | 2066 | 2244 | 2382 | 2052 | 2204 | 2298 |
| 30 | 168 | 362 | 145 | 355 | 391 | 426 | 303 | 135 |
|  |  |  |  |  |  |  |  |  |
|  |  |  |  |  |  |  |  |  |
|  |  |  |  |  |  |  |  |  |
|  |  |  |  |  |  |  |  |  |
| **Co_2305_E_KZH** | **Co_2379_N_KZH** | **Co_2382_S_KZH** | **Co_2385_S_KZH** | **Co_2388_S_KZH** | **Co_2389_N_KZH** | **Co_2392_N_KZH** | **Co_2394_N_KZH** | **Co_1981_Altai** |
|  |  |  |  |  |  |  |  |  |
| 16 | 100 | 206 | 79 | 203 | 226 | 238 | 166 | 77 |
| 14 | 69 | 156 | 66 | 152 | 165 | 188 | 137 | 58 |
| 30 | 169 | 362 | 145 | 355 | 391 | 426 | 303 | 135 |
| 0 | 83 | 73 | 86 | 105 | 110 | 68 | 101 | 61 |
| 16 | 100 | 206 | 79 | 203 | 226 | 238 | 166 | 77 |
| 14 | 69 | 156 | 66 | 152 | 165 | 188 | 137 | 58 |
| 30 | 168 | 362 | 145 | 355 | 391 | 426 | 303 | 135 |
| 3 | 7 | 44 | 12 | 25 | 16 | 52 | 36 | 9 |
| 0 | 83 | 73 | 86 | 105 | 110 | 68 | 101 | 61 |
| 30 | 68,556 | 162,222 | 63,133 | 170,356 | 215,089 | 200,733 | 167,089 | 75,356 |
|  |  |  |  |  |  |  |  |  |
| 30 | 59,38561 | 127,96185 | 51,25544 | 125,48745 | 138,21293 | 150,58493 | 107,10619 | 47,72058 |
| 21,56386 | 24,22069 | 51,76845 | 20,95446 | 50,77449 | 55,88629 | 60,85607 | 43,39072 | 19,53431 |
| 30 | 68,55556 | 162,22222 | 63,13333 | 170,35556 | 215,08889 | 200,73333 | 167,08889 | 75,35556 |
| 30,4959 | 36,59577 | 86,07319 | 33,73149 | 90,36938 | 113,99843 | 106,41554 | 88,64386 | 40,18782 |
|  |  |  |  |  |  |  |  |  |
| 15,00 | 16,80 | 36,20 | 14,50 | 35,50 | 39,10 | 42,60 | 30,30 | 13,50 |
| 1,50 | 0,70 | 4,40 | 1,20 | 2,50 | 1,60 | 5,20 | 3,60 | 0,90 |

| **Co_1982_Altai** | **Co_1983_Altai** | **Co_1984_Altai** | **Co_2296_E_KZH** | **Co_2300_E_KZH** | **Co_2373_N_KZH** | **Mean** | **s.d.** |  |
| --- | --- | --- | --- | --- | --- | --- | --- | --- |
|  |  |  |  |  |  |  |  |  |
| 10 | 10 | 6 | 10 | 10 | 10 | 8,105 | 3,01 |  |
| 4644 | 4644 | 4644 | 4644 | 4644 | 4644 | 4644 | 0 |  |
| 2597 | 2790 | 2847 | 2681 | 2470 | 2924 | 2614 | 615,026 |  |
| 97 | 111 | 127 | 204 | 304 | 333 | 225,263 | 147,575 |  |
|  |  |  |  |  |  |  |  |  |
|  |  |  |  |  |  |  |  |  |
|  |  |  |  |  |  |  |  |  |
|  |  |  |  |  |  |  |  |  |
| **Co_1982_Altai** | **Co_1983_Altai** | **Co_1984_Altai** | **Co_2296_E_KZH** | **Co_2300_E_KZH** | **Co_2373_N_KZH** | **Mean** | **s.d.** |  |
|  |  |  |  |  |  |  |  |  |
| 40 | 63 | 71 | 112 | 162 | 193 | 124,544 | 82,678 |  |
| 58 | 48 | 56 | 92 | 143 | 140 | 101,000 | 66,505 |  |
| 98 | 111 | 127 | 204 | 305 | 333 | 225,544 | 147,734 |  |
| 109 | 106 | 109 | 93 | 114 | 131 | 87,211 | 45,390 |  |
| 39 | 63 | 71 | 112 | 161 | 193 | 124,456 | 82,627 |  |
| 58 | 48 | 56 | 92 | 143 | 140 | 101,000 | 66,505 |  |
| 97 | 111 | 127 | 204 | 304 | 333 | 225,263 | 147,575 | Total: 3801 |
| 19 | 5 | 15 | 35 | 22 | 20 | 23,298 | 26,947 |  |
| 109 | 106 | 109 | 93 | 114 | 131 | 87,211 | 45,39 |  |
| 67,133 | 90,244 | 112,533 | 121,067 | 166,867 | 185,111 | 131,50904 | 69,70466 |  |
|  |  |  |  |  |  |  |  |  |
| 34,28812 | 39,23692 | 55,62044 | 72,1111 | 107,45967 | 117,71076 | 88,0773 | 51,14102 |  |
| 14,13732 | 16,12579 | 26,40317 | 29,33286 | 43,53272 | 47,65059 | 39,21383 | 21,73832 |  |
| 67,13333 | 90,24444 | 112,53333 | 121,06667 | 166,86667 | 185,11111 | 131,50904 | 69,70466 |  |
| 35,84448 | 48,05267 | 65,37405 | 64,33386 | 88,52648 | 98,16357 | 75,03942 | 37,79285 |  |
|  |  |  |  |  |  |  |  |  |
| 9,70 | 11,10 | 21,17 | 20,40 | 30,40 | 33,30 |  |  |  |
| 1,90 | 0,50 | 2,50 | 3,50 | 2,20 | 2,00 |  |  |  |

**Supplementary File 12-2: Molecular diversity indexes analysis of five genetic clusters**, each consisting of five different populations.

| **Statistics** |  |  |  | **Altai** | **S_KZH** | **E_KZH** | **Mongolia** | **N_KZH** | **Mean** | **s.d.** |  |
| --- | --- | --- | --- | --- | --- | --- | --- | --- | --- | --- | --- |
|  |  |  |  |  |  |  |  |  |  |  |  |
| **No.** | **of** | **gene** | **copies** | 38 | 50 | 36 | 42 | 44 | 42.000 | 5.477 |  |
| **No.** | **of** | **loci** |  | 4644 | 4644 | 4644 | 4644 | 4644 | 4.644.000 | 0.000 |  |
| **No.** | **of** | **usable** | **loci** | 1579 | 1724 | 1817 | 1994 | 1921 | 1.807.000 | 163.507 |  |
| **No.** | **of** | **polym.** | **loci** | 442 | 710 | 364 | 307 | 639 | 492.400 | 174.815 |  |
|  |  |  |  |  |  |  |  |  |  |  |  |
|  |  |  |  |  |  |  |  |  |  |  |  |
|  |  |  |  |  |  |  |  |  |  |  |  |
| **Statistics** |  |  |  | **Altai** | **S_KZH** | **E_KZH** | **Mongolia** | **N_KZH** | **Mean** | **s.d.** |  |
|  |  |  |  |  |  |  |  |  |  |  |  |
| **No.** | **of** | **transitions** |  | 242 | 387 | 190 | 160 | 360 | 267,80 | 101,30 |  |
| **No.** | **of** | **transversions** |  | 201 | 325 | 174 | 147 | 280 | 225,40 | 74,64 |  |
| **No.** | **of** | **substitutions** |  | 443 | 712 | 364 | 307 | 640 | 493,20 | 175,58 |  |
| **No.** | **of** | **indels** |  | 71 | 99 | 66 | 127 | 125 | 97,60 | 28,82 |  |
| **No.** | **of** | **ts.** | **sites** | 242 | 385 | 190 | 160 | 359 | 267,20 | 100,49 |  |
| **No.** | **of** | **tv.** | **sites** | 201 | 325 | 174 | 147 | 280 | 225,40 | 74,64 |  |
| **No.** | **of** | **subst.** | **sites** | 442 | 710 | 364 | 307 | 639 | 492,40 | 174,82 | Total: 1332,00 |
| **No.** | **private** | **subst.** | **sites** | 89 | 264 | 78 | 62 | 150 | 128,60 | 82,70 |  |
| **No.** | **of** | **indel** | **sites** | 71 | 99 | 66 | 127 | 125 | 97,60 | 28,82 |  |
| **Pi** |  |  |  | 124,15 | 169,36 | 109,69 | 111,79 | 165,17 | 136,03 | 29,08 |  |
|  |  |  |  |  |  |  |  |  |  |  |  |
| **Theta_S** |  |  |  | 105,20 | 158,51 | 87,78 | 71,35 | 146,90 | 113,95 | 37,58 |  |
| **s.d.** | **Theta_S** |  |  | 30,86 | 43,76 | 26,11 | 20,62 | 41,65 | 32,60 | 9,94 |  |
| **Theta_pi** |  |  |  | 124,15 | 169,36 | 109,69 | 111,79 | 165,17 | 136,03 | 29,08 |  |
| **s.d.** | **Theta_pi** |  |  | 60,51 | 81,86 | 53,58 | 54,37 | 80,07 | 66,08 | 13,86 |  |

**Supplementary File 12-3: Pairwise F_ST_ population values** of investigated *Capsella orientalis* populations. F_ST_ values greater than 0.50 are labelled in red and F_ST_ values below 0.10 are labelled in light blue.

|  | **Co_2380_N_KZH** | **Co_2386_S_KZH** | **Co2397_Altai** | **Co_2400_Altai** | **Co_1980_Altai** | **Co_1933_Mongolia** | **Co2374_N_KZH** | **Co_2381_S_KZH** | **Co_2346_N_KZH** | **Co_2223_Mongolia** | **Co_1985_Altai** | **Co_26144_Mongolia** | **Co_2396_Altai** | **Co_2387_S_KZH** | **Co_2343_S_KZH** | **Co_2349_N_KZH** | **Co_2393_N_KZH** | **Co_2395_S_KZH** | **Co_2391_N_KZH** |
| --- | --- | --- | --- | --- | --- | --- | --- | --- | --- | --- | --- | --- | --- | --- | --- | --- | --- | --- | --- |
| **Co_2380_N_KZH** | 0,00000 |  |  |  |  |  |  |  |  |  |  |  |  |  |  |  |  |  |  |
| **Co_2386_S_KZH** | 0,13945 | 0,00000 |  |  |  |  |  |  |  |  |  |  |  |  |  |  |  |  |  |
| **Co2397_Altai** | 0,23767 | 0,21997 | 0,00000 |  |  |  |  |  |  |  |  |  |  |  |  |  |  |  |  |
| **Co_2400_Altai** | 0,20106 | 0,16150 | 0,16864 | 0,00000 |  |  |  |  |  |  |  |  |  |  |  |  |  |  |  |
| **Co_1980_Altai** | 0,27195 | 0,22933 | 0,36461 | 0,30909 | 0,00000 |  |  |  |  |  |  |  |  |  |  |  |  |  |  |
| **Co_1933_Mongolia** | 0,22973 | 0,16874 | 0,28234 | 0,20017 | 0,28019 | 0,00000 |  |  |  |  |  |  |  |  |  |  |  |  |  |
| **Co2374_N_KZH** | 0,14920 | 0,11911 | 0,18397 | 0,13519 | 0,26386 | 0,18488 | 0,00000 |  |  |  |  |  |  |  |  |  |  |  |  |
| **Co_2381_S_KZH** | 0,14026 | 0,11808 | 0,22889 | 0,17170 | 0,24204 | 0,17866 | 0,12406 | 0,00000 |  |  |  |  |  |  |  |  |  |  |  |
| **Co_2346_N_KZH** | 0,15024 | 0,12895 | 0,17973 | 0,14378 | 0,26908 | 0,17879 | 0,09224 | 0,11852 | 0,00000 |  |  |  |  |  |  |  |  |  |  |
| **Co_2223_Mongolia** | 0,25570 | 0,21316 | 0,28447 | 0,26323 | 0,33912 | 0,27556 | 0,24785 | 0,25965 | 0,25519 | 0,00000 |  |  |  |  |  |  |  |  |  |
| **Co_1985_Altai** | 0,18817 | 0,16834 | 0,18026 | 0,13145 | 0,28280 | 0,21707 | 0,13267 | 0,17534 | 0,15153 | 0,24996 | 0,00000 |  |  |  |  |  |  |  |  |
| **Co_26144_Mongolia** | 0,26113 | 0,21179 | 0,26055 | 0,19178 | 0,36894 | 0,15245 | 0,19606 | 0,22645 | 0,19524 | 0,29670 | 0,22327 | 0,00000 |  |  |  |  |  |  |  |
| **Co_2396_Altai** | 0,22388 | 0,21593 | 0,18627 | 0,20774 | 0,34364 | 0,25802 | 0,16740 | 0,20005 | 0,15829 | 0,33571 | 0,21397 | 0,27344 | 0,00000 |  |  |  |  |  |  |
| **Co_2387_S_KZH** | 0,18383 | 0,13556 | 0,23308 | 0,18127 | 0,29618 | 0,20026 | 0,14492 | 0,15541 | 0,15273 | 0,24820 | 0,18962 | 0,21624 | 0,23124 | 0,00000 |  |  |  |  |  |
| **Co_2343_S_KZH** | 0,35923 | 0,31710 | 0,50062 | 0,46247 | 0,33253 | 0,35738 | 0,35377 | 0,30076 | 0,35128 | 0,43741 | 0,41610 | 0,47589 | 0,44653 | 0,37952 | 0,00000 |  |  |  |  |
| **Co_2349_N_KZH** | 0,24581 | 0,22024 | 0,31191 | 0,30253 | 0,25047 | 0,28219 | 0,24825 | 0,25956 | 0,25476 | 0,22693 | 0,25280 | 0,32199 | 0,33616 | 0,28558 | 0,28522 | 0,00000 |  |  |  |
| **Co_2393_N_KZH** | 0,16693 | 0,13843 | 0,18832 | 0,17381 | 0,26076 | 0,21485 | 0,14808 | 0,18316 | 0,15756 | 0,15888 | 0,13941 | 0,21939 | 0,23703 | 0,18609 | 0,37111 | 0,14672 | 0,00000 |  |  |
| **Co_2395_S_KZH** | 0,28252 | 0,23188 | 0,41024 | 0,35996 | 0,25799 | 0,27333 | 0,26755 | 0,22638 | 0,25034 | 0,35390 | 0,32924 | 0,37843 | 0,35980 | 0,29006 | 0,21541 | 0,26520 | 0,29166 | 0,00000 |  |
| **Co_2391_N_KZH** | 0,17285 | 0,16059 | 0,21203 | 0,16881 | 0,30512 | 0,23533 | 0,12982 | 0,17265 | 0,14155 | 0,25526 | 0,15670 | 0,24930 | 0,22909 | 0,19287 | 0,42766 | 0,22970 | 0,13245 | 0,33998 | 0,00000 |
| **Co_2390_N_KZH** | 0,12781 | 0,09389 | 0,19887 | 0,15259 | 0,21475 | 0,16690 | 0,08494 | 0,10367 | 0,09132 | 0,21099 | 0,13372 | 0,20697 | 0,18199 | 0,14428 | 0,29425 | 0,18841 | 0,11721 | 0,20701 | 0,11300 |
| **Co_2347_N_KZH** | 0,30892 | 0,29499 | 0,43134 | 0,41071 | 0,31854 | 0,36682 | 0,32049 | 0,29793 | 0,32710 | 0,36718 | 0,35686 | 0,45907 | 0,42055 | 0,36180 | 0,33963 | 0,18793 | 0,27733 | 0,30645 | 0,33791 |
| **Co_26073_Mongolia** | 0,39339 | 0,33934 | 0,50716 | 0,45407 | 0,37470 | 0,28897 | 0,37951 | 0,33563 | 0,37909 | 0,43671 | 0,42100 | 0,41547 | 0,47630 | 0,39160 | 0,30533 | 0,28302 | 0,36644 | 0,30641 | 0,43980 |
| **Co_2345_S_KZH** | 0,20255 | 0,16727 | 0,31814 | 0,27337 | 0,23424 | 0,22192 | 0,19796 | 0,15848 | 0,19620 | 0,26231 | 0,24683 | 0,29545 | 0,28140 | 0,21969 | 0,18551 | 0,21330 | 0,20890 | 0,19081 | 0,24386 |
| **Co_2383_S_KZH** | 0,20563 | 0,15474 | 0,25346 | 0,22088 | 0,29626 | 0,22915 | 0,19061 | 0,20004 | 0,20363 | 0,20613 | 0,20176 | 0,25248 | 0,29055 | 0,18849 | 0,39516 | 0,22727 | 0,14093 | 0,30697 | 0,21565 |
| **Co_2384_S_KZH** | 0,22784 | 0,15044 | 0,33322 | 0,28734 | 0,25353 | 0,23010 | 0,20431 | 0,19209 | 0,21739 | 0,25767 | 0,25815 | 0,30471 | 0,32245 | 0,21478 | 0,30007 | 0,21098 | 0,19581 | 0,19692 | 0,27101 |
| **Co_2303_E_KZH** | 0,22459 | 0,19026 | 0,23042 | 0,20652 | 0,33138 | 0,24084 | 0,18875 | 0,19845 | 0,18429 | 0,24699 | 0,21392 | 0,23948 | 0,25035 | 0,21174 | 0,43804 | 0,30476 | 0,19013 | 0,34678 | 0,23299 |
| **Co_2329_S_KZH** | 0,17093 | 0,13159 | 0,23379 | 0,19364 | 0,23387 | 0,17549 | 0,13463 | 0,13960 | 0,13252 | 0,21436 | 0,18807 | 0,20012 | 0,21796 | 0,16078 | 0,26712 | 0,22004 | 0,15679 | 0,19595 | 0,20212 |
| **Co_2379_N_KZH** | 0,16190 | 0,17588 | 0,26545 | 0,22408 | 0,29201 | 0,24531 | 0,16270 | 0,16114 | 0,16363 | 0,29884 | 0,19090 | 0,28788 | 0,24686 | 0,21832 | 0,37265 | 0,26616 | 0,19982 | 0,28656 | 0,20193 |
| **Co_2382_S_KZH** | 0,15596 | 0,13413 | 0,22109 | 0,19760 | 0,24610 | 0,20389 | 0,15339 | 0,15258 | 0,15607 | 0,18623 | 0,16161 | 0,22621 | 0,24564 | 0,17992 | 0,32823 | 0,18271 | 0,10626 | 0,24914 | 0,16663 |
| **Co_2385_S_KZH** | 0,21705 | 0,16019 | 0,31625 | 0,27892 | 0,27500 | 0,22730 | 0,21025 | 0,19198 | 0,21203 | 0,25802 | 0,24895 | 0,28427 | 0,31169 | 0,21972 | 0,30339 | 0,22901 | 0,18740 | 0,24023 | 0,24880 |
| **Co_2388_S_KZH** | 0,14253 | 0,11177 | 0,22341 | 0,17473 | 0,22700 | 0,17946 | 0,12506 | 0,12394 | 0,13033 | 0,20809 | 0,16316 | 0,21404 | 0,22145 | 0,13324 | 0,29170 | 0,20979 | 0,13691 | 0,22591 | 0,14677 |
| **Co_2389_N_KZH** | 0,15017 | 0,12186 | 0,21207 | 0,17423 | 0,23637 | 0,19098 | 0,11840 | 0,12356 | 0,12607 | 0,22652 | 0,15700 | 0,23109 | 0,19692 | 0,16282 | 0,32505 | 0,21621 | 0,14055 | 0,24374 | 0,14120 |
| **Co_2392_N_KZH** | 0,14741 | 0,11376 | 0,23872 | 0,20003 | 0,20599 | 0,18229 | 0,14341 | 0,13882 | 0,14430 | 0,19273 | 0,17591 | 0,24430 | 0,23606 | 0,17425 | 0,27699 | 0,16270 | 0,11876 | 0,19157 | 0,16209 |
| **Co_2394_N_KZH** | 0,16571 | 0,12481 | 0,26291 | 0,21002 | 0,19457 | 0,18561 | 0,16113 | 0,14636 | 0,16031 | 0,21858 | 0,19385 | 0,25570 | 0,25705 | 0,19355 | 0,28028 | 0,17373 | 0,14228 | 0,19351 | 0,16951 |
| **Co_1981_Altai** | 0,18060 | 0,14799 | 0,22565 | 0,15980 | 0,20038 | 0,20712 | 0,14088 | 0,15430 | 0,15412 | 0,25893 | 0,12554 | 0,26198 | 0,22660 | 0,19896 | 0,34453 | 0,20658 | 0,15638 | 0,26067 | 0,15673 |
| **Co_1982_Altai** | 0,22417 | 0,20256 | 0,24082 | 0,17518 | 0,29897 | 0,24807 | 0,15013 | 0,18664 | 0,16142 | 0,33829 | 0,14955 | 0,28748 | 0,22449 | 0,22846 | 0,41479 | 0,29770 | 0,22234 | 0,32806 | 0,19656 |
| **Co_1983_Altai** | 0,22530 | 0,19715 | 0,25530 | 0,18192 | 0,28994 | 0,23881 | 0,15062 | 0,17187 | 0,15473 | 0,36600 | 0,16565 | 0,28708 | 0,22755 | 0,23127 | 0,41142 | 0,31845 | 0,24533 | 0,32375 | 0,21267 |
| **Co_2296_E_KZH** | 0,22862 | 0,20331 | 0,30863 | 0,26173 | 0,27547 | 0,21502 | 0,20772 | 0,17348 | 0,19112 | 0,30591 | 0,26408 | 0,28614 | 0,26434 | 0,24067 | 0,31441 | 0,29947 | 0,25215 | 0,25267 | 0,26817 |
| **Co_2300_E_KZH** | 0,22160 | 0,18758 | 0,32300 | 0,28103 | 0,24721 | 0,22276 | 0,22530 | 0,18196 | 0,21080 | 0,26889 | 0,26247 | 0,30887 | 0,29754 | 0,24179 | 0,29043 | 0,24685 | 0,22214 | 0,21492 | 0,26442 |
| **Co_2373_N_KZH** | 0,19744 | 0,17910 | 0,27583 | 0,21407 | 0,29089 | 0,22831 | 0,11782 | 0,14419 | 0,12825 | 0,35417 | 0,20899 | 0,27968 | 0,20973 | 0,21269 | 0,34808 | 0,29707 | 0,23584 | 0,27319 | 0,19594 |

|  | **Co_2390_N_KZH** | **Co_2347_N_KZH** | **Co_26073_Mongolia** | **Co_2345_S_KZH** | **Co_2383_S_KZH** | **Co_2384_S_KZH** | **Co_2303_E_KZH** | **Co_2329_S_KZH** | **Co_2379_N_KZH** | **Co_2382_S_KZH** | **Co_2385_S_KZH** | **Co_2388_S_KZH** | **Co_2389_N_KZH** | **Co_2392_N_KZH** | **Co_2394_N_KZH** | **Co_1981_Altai** | **Co_1982_Altai** | **Co_1983_Altai** | **Co_2296_E_KZH** | **Co_2300_E_KZH** | **Co_2373_N_KZH** |
| --- | --- | --- | --- | --- | --- | --- | --- | --- | --- | --- | --- | --- | --- | --- | --- | --- | --- | --- | --- | --- | --- |
| **Co_2390_N_KZH** | 0,00000 |  |  |  |  |  |  |  |  |  |  |  |  |  |  |  |  |  |  |  |  |
| **Co_2347_N_KZH** | 0,25334 | 0,00000 |  |  |  |  |  |  |  |  |  |  |  |  |  |  |  |  |  |  |  |
| **Co_26073_Mongolia** | 0,31917 | 0,40352 | 0,00000 |  |  |  |  |  |  |  |  |  |  |  |  |  |  |  |  |  |  |
| **Co_2345_S_KZH** | 0,14745 | 0,23816 | 0,26594 | 0,00000 |  |  |  |  |  |  |  |  |  |  |  |  |  |  |  |  |  |
| **Co_2383_S_KZH** | 0,16739 | 0,34171 | 0,40045 | 0,22942 | 0,00000 |  |  |  |  |  |  |  |  |  |  |  |  |  |  |  |  |
| **Co_2384_S_KZH** | 0,16478 | 0,29405 | 0,33362 | 0,18825 | 0,18894 | 0,00000 |  |  |  |  |  |  |  |  |  |  |  |  |  |  |  |
| **Co_2303_E_KZH** | 0,19128 | 0,40915 | 0,45483 | 0,25123 | 0,22684 | 0,28175 | 0,00000 |  |  |  |  |  |  |  |  |  |  |  |  |  |  |
| **Co_2329_S_KZH** | 0,12236 | 0,29548 | 0,30588 | 0,13708 | 0,17636 | 0,16224 | 0,17495 | 0,00000 |  |  |  |  |  |  |  |  |  |  |  |  |  |
| **Co_2379_N_KZH** | 0,15373 | 0,33179 | 0,40876 | 0,22178 | 0,23124 | 0,24366 | 0,25188 | 0,19311 | 0,00000 |  |  |  |  |  |  |  |  |  |  |  |  |
| **Co_2382_S_KZH** | 0,12059 | 0,26694 | 0,34383 | 0,17196 | 0,13118 | 0,16872 | 0,19754 | 0,14783 | 0,16185 | 0,00000 |  |  |  |  |  |  |  |  |  |  |  |
| **Co_2385_S_KZH** | 0,17352 | 0,29995 | 0,33825 | 0,18287 | 0,17991 | 0,15701 | 0,26196 | 0,16905 | 0,22357 | 0,14743 | 0,00000 |  |  |  |  |  |  |  |  |  |  |
| **Co_2388_S_KZH** | 0,10002 | 0,26850 | 0,31944 | 0,14279 | 0,15066 | 0,16245 | 0,19643 | 0,13601 | 0,15695 | 0,11149 | 0,14336 | 0,00000 |  |  |  |  |  |  |  |  |  |
| **Co_2389_N_KZH** | 0,08515 | 0,27178 | 0,34865 | 0,16783 | 0,18184 | 0,19019 | 0,19878 | 0,14783 | 0,17146 | 0,13589 | 0,19617 | 0,12105 | 0,00000 |  |  |  |  |  |  |  |  |
| **Co_2392_N_KZH** | 0,09128 | 0,22580 | 0,30059 | 0,15241 | 0,14879 | 0,13736 | 0,21354 | 0,13800 | 0,17203 | 0,11252 | 0,14763 | 0,11114 | 0,11966 | 0,00000 |  |  |  |  |  |  |  |
| **Co_2394_N_KZH** | 0,10624 | 0,22975 | 0,29959 | 0,14813 | 0,17377 | 0,15331 | 0,23521 | 0,14850 | 0,18748 | 0,13515 | 0,16557 | 0,12487 | 0,12807 | 0,08852 | 0,00000 |  |  |  |  |  |  |
| **Co_1981_Altai** | 0,11003 | 0,28128 | 0,36086 | 0,20589 | 0,20988 | 0,21409 | 0,24338 | 0,18001 | 0,18896 | 0,16451 | 0,22956 | 0,14600 | 0,13719 | 0,12810 | 0,13255 | 0,00000 |  |  |  |  |  |
| **Co_1982_Altai** | 0,15516 | 0,38512 | 0,44724 | 0,26455 | 0,27953 | 0,29784 | 0,26828 | 0,21655 | 0,21433 | 0,22495 | 0,28912 | 0,19045 | 0,17730 | 0,21206 | 0,21661 | 0,14881 | 0,00000 |  |  |  |  |
| **Co_1983_Altai** | 0,16255 | 0,39942 | 0,44929 | 0,26344 | 0,29747 | 0,30782 | 0,28351 | 0,21327 | 0,22702 | 0,24503 | 0,30455 | 0,20180 | 0,18481 | 0,22029 | 0,22170 | 0,14143 | 0,15081 | 0,00000 |  |  |  |
| **Co_2296_E_KZH** | 0,17746 | 0,35748 | 0,35834 | 0,17780 | 0,28280 | 0,26316 | 0,19280 | 0,15758 | 0,25524 | 0,22594 | 0,25265 | 0,19518 | 0,19816 | 0,20153 | 0,21099 | 0,23148 | 0,26418 | 0,25965 | 0,00000 |  |  |
| **Co_2300_E_KZH** | 0,17553 | 0,30310 | 0,32760 | 0,17466 | 0,24073 | 0,20826 | 0,18335 | 0,16955 | 0,24136 | 0,19575 | 0,21223 | 0,17815 | 0,18999 | 0,15764 | 0,16541 | 0,21298 | 0,27686 | 0,28878 | 0,14994 | 0,00000 |  |
| **Co_2373_N_KZH** | 0,12573 | 0,35928 | 0,40737 | 0,21488 | 0,28301 | 0,27380 | 0,26300 | 0,18380 | 0,20110 | 0,22036 | 0,26856 | 0,17786 | 0,16475 | 0,20085 | 0,20437 | 0,18249 | 0,18129 | 0,14726 | 0,21084 | 0,24929 | 0,00000 |

**Supplementary File 12-4: F_ST_ analysis of five genetic clusters**, each consisting of five different populations. The values in bold represent the highest F_ST_ and the value in cursive represent the lowest F_ST_ value.

| **Distance** | **method:** | **No.** | **of** | **different** | **alleles** | **(FST)** |
| --- | --- | --- | --- | --- | --- | --- |
|  |  |  |  |  |  |  |
|  |  | **Altai** | **S_KZH** | **E_KZH** | **Mongolia** | **N_KZH** |
|  | **Altai** | 0.00000 |  |  |  |  |
|  | **S_KZH** | 0.06173 | 0.00000 |  |  |  |
|  | **E_KZH** | **0.11494** | 0.07515 | 0.00000 |  |  |
|  | **Mongolia** | 0.09733 | 0.06720 | **0.11331** | 0.00000 |  |
|  | **N_KZH** | 0.04805 | *0.03912* | 0.10146 | 0.09187 | 0.00000 |

**Supplementary File 12-5: AMOVA results of five genetic clusters**, each consisting of five different populations (left) and significance test (right).

| **Source** | **of** | **Sum** | **of** | **Variance** | **Percentage** |
| --- | --- | --- | --- | --- | --- |
| **variation** | **d.f.** | **squares** | **components** | **of** | **variation** |
|  |  |  |  |  |  |
| **Among** |  |  |  |  |  |
| **groups** | 4 | 14838,84 | 32,69 | Va | 3,70 |
|  |  |  |  |  |  |
| **Among** |  |  |  |  |  |
| **populations** |  |  |  |  |  |
| **within** |  |  |  |  |  |
| **groups** | 20 | 44996,39 | 93,74 | Vb | 10,60 |
|  |  |  |  |  |  |
| **Among** |  |  |  |  |  |
| **individuals** |  |  |  |  |  |
| **within** |  |  |  |  |  |
| **populations** | 80 | 118475,95 | 723,40 | Vc | 81,83 |
| **Within** |  |  |  |  |  |
| **individuals** | 105 | 3586,50 | 34,16 | Vd | 3,86 |
|  |  |  |  |  |  |
| **Total** | 209 | 181897,686 | 883,98193 |  |  |
|  |  |  |  |  |  |
| **Fixation** | **Indices** |  |  |  |  |
| **FIS** | : | 0,95491 |  |  |  |
| **FSC** | : | 0,11012 |  |  |  |
| **FCT** | : | 0,03698 |  |  |  |
| **FIT** | : | 0,96136 |  |  |  |

| **Significance** | **tests** | **(1023** | **permutations)** |  |  |  |  |  |  |  |
| --- | --- | --- | --- | --- | --- | --- | --- | --- | --- | --- |
| ------------------ |  |  |  |  |  |  |  |  |  |  |
|  |  |  |  |  |  |  |  |  |  |  |
| Vd | and | FIT | : | P(rand, | value | &lt; | obs, | value) | = | 0 |
|  | P(rand, | value | = | obs, | value) | = | 0 |  |  |  |
|  | P-value | = | 0,000+-0,000 |  |  |  |  |  |  |  |
|  |  |  |  |  |  |  |  |  |  |  |
| Vc | and | FIS | : | P(rand, | value | > | obs, | value) | = | 0 |
|  | P(rand, | value | = | obs, | value) | = | 0 |  |  |  |
|  | P-value | = | 0,000+-0,000 |  |  |  |  |  |  |  |
|  |  |  |  |  |  |  |  |  |  |  |
| Vb | and | FSC | : | P(rand, | value | > | obs, | value) | = | 0 |
|  | P(rand, | value | = | obs, | value) | = | 0 |  |  |  |
|  | P-value | = | 0,000+-0,000 |  |  |  |  |  |  |  |
|  |  |  |  |  |  |  |  |  |  |  |
| Va | and | FCT | : | P(rand, | value | > | obs, | value) | = | 0,00293 |
|  | P(rand, | value | = | obs, | value) | = | 0 |  |  |  |
|  | P-value | = | 0,003+-0,002 |  |  |  |  |  |  |  |

**Supplementary File 12-6: Observed (H_O_) and expected (H_E_) heterozygosity values** of populations consisting of more than one accession with corresponding standard deviations (s.d.). In bold are the averaged values of each parameter.

| **Population** | **Ho** | **s.d.** | **He** | **s.d.** |
| --- | --- | --- | --- | --- |
| CO2380 | 0,08158 | 0,17334 | 0,5231 | 0,17876 |
| CO2386 | 0,07036 | 0,17523 | 0,53858 | 0,16838 |
| CO2397 | 0,07897 | 0,19213 | 0,53064 | 0,16301 |
| CO2400 | 0,09167 | 0,17822 | 0,51601 | 0,1778 |
| CO1980 | 0,10548 | 0,27936 | 0,54917 | 0,20071 |
| CO1979 | 0,13101 | 0,26368 | 0,59478 | 0,12431 |
| CO1933 | 0,08518 | 0,19642 | 0,53322 | 0,17797 |
| CO2374 | 0,06458 | 0,16259 | 0,51258 | 0,17228 |
| CO2381 | 0,09193 | 0,17564 | 0,49444 | 0,187 |
| CO2348 | 0,03215 | 0,13643 | 0,56111 | 0,08186 |
| CO2346 | 0,06393 | 0,15026 | 0,49492 | 0,16416 |
| CO2289 | 0,06212 | 0,18534 | 0,61948 | 0,11218 |
| CO2223 | 0,07008 | 0,1941 | 0,5532 | 0,17083 |
| CO1985 | 0,05615 | 0,16502 | 0,53508 | 0,17053 |
| CO26144 | 0,07074 | 0,18093 | 0,56239 | 0,14855 |
| CO2396 | 0,04911 | 0,14601 | 0,49696 | 0,17044 |
| CO2387 | 0,05474 | 0,14845 | 0,5153 | 0,1826 |
| CO2372 | 0,08419 | 0,257 | 0,68786 | 0,08541 |
| CO2370 | 0,03232 | 0,14768 | 0,66984 | 0,04353 |
| CO2358 | 0,0444 | 0,18387 | 0,67407 | 0,05771 |
| CO2343 | 0,05932 | 0,12661 | 0,46531 | 0,14895 |
| CO2349 | 0,01806 | 0,10443 | 0,52615 | 0,14669 |
| CO2391 | 0,05106 | 0,14426 | 0,5268 | 0,16553 |
| CO2391 | 0,07745 | 0,15463 | 0,49457 | 0,16666 |
| CO2393 | 0,00921 | 0,06761 | 0,50988 | 0,10125 |
| CO2395 | 0,15876 | 0,29825 | 0,68289 | 0,10112 |
| CO2390 | 0,05717 | 0,16166 | 0,51153 | 0,16686 |
| CO2347 | 0,01437 | 0,08459 | 0,542 | 0,13878 |
| CO26073 | 0,03134 | 0,14632 | 0,52322 | 0,13272 |
| CO26082 | 0,04596 | 0,18098 | 0,59814 | 0,10393 |
| CO2345 | 0,06134 | 0,17272 | 0,5117 | 0,17173 |
| CO2383 | 0,03899 | 0,1342 | 0,55164 | 0,16639 |
| CO2384 | 0,03815 | 0,14976 | 0,56918 | 0,147 |
| CO2292 | 0,06147 | 0,19054 | 0,61728 | 0,11174 |
| CO2303 | 0,04694 | 0,15041 | 0,5354 | 0,17172 |
| CO2329 | 0,0659 | 0,16069 | 0,50794 | 0,17081 |
| CO2379 | 0,07136 | 0,16638 | 0,51497 | 0,18562 |
| CO2382 | 0,06262 | 0,15294 | 0,53507 | 0,1711 |
| CO2385 | 0,05642 | 0,16034 | 0,52625 | 0,18443 |
| CO2388 | 0,08367 | 0,16681 | 0,52492 | 0,17459 |
| CO2389 | 0,05796 | 0,15467 | 0,54134 | 0,16883 |
| CO2392 | 0,04703 | 0,17241 | 0,53637 | 0,17164 |
| CO2394 | 0,05796 | 0,17884 | 0,54601 | 0,16913 |
| CO1981 | 0,05437 | 0,16448 | 0,53952 | 0,16903 |
| CO1982 | 0,05644 | 0,15711 | 0,52051 | 0,1749 |
| CO1983 | 0,05389 | 0,1536 | 0,50083 | 0,16417 |
| CO1984 | 0,07355 | 0,20219 | 0,62382 | 0,11777 |
| CO2296 | 0,04585 | 0,15019 | 0,50332 | 0,15816 |
| CO2300 | 0,4994 | 0,17279 | 0,53777 | 0,17652 |
| CO2373 | 0,07059 | 0,15746 | 0,48853 | 0,16143 |
| **Average** | **0,071** | **0,169** | **0,546** | **0,152** |
